# Toward a Common Framework for the Design of Soft Robotic Manipulators with Fluidic Actuation

**DOI:** 10.1089/soro.2017.0105

**Published:** 2018-10-10

**Authors:** Arnau Garriga-Casanovas, Ian Collison, Ferdinando Rodriguez y Baena

**Affiliations:** ^1^ Mechatronics in Medicine Laboratory, Department of Mechanical Engineering, Imperial College London, London, United Kingdom.; ^2^ Rolls-Royce, plc, London, United Kingdom.

**Keywords:** soft robots with fluidic actuation, common design framework, soft robotic manipulators

## Abstract

Soft robotic manipulators with fluidic actuation are devices with easily deformable structures that comprise a set of chambers that can be pressurized to achieve structural deflection. These devices have experienced a rapid development in recent years, which is not least due to the advantages they offer in terms of robustness, affordability, and compliance. Nowadays, however, soft robotic manipulators are designed mostly by intuition, which complicates design improvement and hampers the advancement of the field. In this article, a general study of the design of soft robotic manipulators with fluidic actuation is presented using an analytical derivation. The study relies on a novel approach that is applicable to a general design and thus provides a common framework for the design of soft robots. In the study, two design layouts of interest are first justified, which correspond to extending and contracting devices. Design principles for each of the layouts are subsequently derived, both for planar and 3D scenarios, and considering operation to support any external loading and to provide any desired deflection. These principles are found to agree with the main design trends in the literature, although they also highlight the potential for improvement in specific aspects of the design geometry and stiffness distribution. The principles are used to identify the most suitable design for both extending and contracting devices in 2D and 3D and extract insight into their behavior. To showcase the use of these design principles, a prototypical scenario in minimally invasive surgery requiring a manipulator segment capable of bending in any direction is defined, where the objective is to maximize its lateral force. The principles are applied to determine the most suitable design. These also highlight the need for numerical analysis to optimize two design parameters. Finite element simulations are developed, and their results are reported. Among the most relevant is the fact that the cross-sectional area with pressurized fluid should be maximized and that the stiffness in the design should be selected to minimize structural stretching. The simulations yield the optimal design, which offers higher force than existing, reference ones. The simulations also provide verification for the study.

## Introduction

Soft robots are commonly defined as devices composed of low-stiffness materials,^1^ which are frequently used to achieve significant structural deformations and displacements. The field of soft robotics has received significant attention in recent years, with a myriad of new devices proposed and developed.^2^ These devices are aimed at a wide range of applications, including minimally invasive surgery (MIS),^3,4^ micro-gripping,^5^ and swimming.^6^

Pressurized fluids are the most common and therefore relevant means of actuation in soft robotics. Soft robots with fluidic actuation are generally used as manipulators,^7^ as limbs for locomotion,^8^ or as actuators in more complex systems such as rehabilitation or assistive devices.^9,10^

The majority of these applications require the soft robot to provide a controlled motion between two points of interest in a solid structure while supporting external forces and moments. Soft robots with fluidic actuation offering this type of operation represent the focus of this work. Specifically, we concentrate on the design of the individual elements providing the controlled motion, which may be part of a system comprising multiple similar elements, such as a manipulator composed of serially stacked segments. Since the design of each of these elements can be studied separately, in this work, the elements are treated as individual devices.

These soft robotic devices can be classified according to the motion they provide between the two points of interest when pressurized. This fact results in three categories: devices that provide elongation, contraction, and bending. The design of elongating devices is relatively straightforward, as the elongation is directly created by the pressure applied to the chamber walls in the elongation direction, and the structure generally opposes to it. Thus, the design simply involves a structure that facilitates elongation while containing the pressurized fluid and preventing radial expansion. In addition, piston-cylinder devices provide efficient solutions to elongation needs,^11^ hence elongating devices are not considered further. Contracting devices are equivalent to pneumatic artificial muscles (PAMs). The design and mechanical properties of PAMs are extensively studied in the literature^12,13^ and therefore are not analyzed further in this work.

The design of devices that provide bending, however, is challenging, and a general rationale for their design is not available. The canonical application for bending devices is manipulation, and therefore, they are generally interpreted as segments of soft robotic manipulators. Due to their relevance in manipulation, a profusion of bending devices has been proposed in recent decades, with a variety of designs. One of the pioneering is the flexible microactuator (FMA),^14,15^ which introduced a design layout that has been subsequently adapted and further investigated by various groups.^16–19^ Another concept that uses three parallel PAMs to achieve bending was incorporated in the segments of the OctArm robot.^20,21^ More recently, bending devices with alternative layouts have been proposed, including PneuNets,^22^ miniature actuators,^23,24^ fingers in a hand,^25^ and bending devices directly applied for the development of manipulators.^7,26^ However, despite the wide range of designs now available, bending devices are still designed mostly by intuition.

A first study of the design of bending devices was recently published.^27^ However, it only offers a specific analysis of a set of predefined designs, but it is not applicable for a generalized design study. In addition, the derivation in Ref.^27^ relies on equilibrium conditions that may not always be justified, and the article only considers the effects of external forces at zero deflection configurations. A comprehensive set of tools for the design of soft robots are available at the soft robotics toolkit.^28^ However, these tools are predominantly based on finite element (FE) methods and experiments centered on a set of predefined designs, which are suitable for the analysis and optimization of specified classes of designs but are not applicable to address the design problem in general. In this regard, to the best of our knowledge, there is no general framework for the design of bending devices, which hinders the identification of the best existing designs, complicates the development of novel and improved devices, and ultimately hampers the advancement of the field.

In this article, a general study of the design of soft robots with fluidic actuation that provides bending is presented. In the study, the design layouts of interest are first justified, and a set of design principles are then derived, which enable subsequent design optimization. The foundation for the study is a novel approach that considers the equilibrium of devices isolated in arbitrary cross sections to provide insight into their mechanical behavior. Such an approach is adapted from existing work on tendon-driven continuum manipulators,^29^ with parallelisms that are apparent in the analysis. The approach serves both to study the design of soft robotic manipulators with fluidic actuation and to mechanically model them for accurate control. In this article, the focus is on design, leaving mechanical modeling for future work.

The approach proposed in this article is applicable to any design, and therefore, the study developed here is general. The findings in terms of design principles coincide with some design trends in the existing literature, elucidating the relevance of this work. In this regard, this article aims to contribute toward the development of a common framework for the design of bending devices, serving as a reference to compare existing designs, and providing an analytical instrument, together with a set of principles for the design of soft robotic manipulators. It should be noted that the nature of the analysis in this work is generally qualitative, although mathematical elements are used to facilitate the derivation.

The article is structured as follows. The specific design problem is formulated in the Problem Formulation section. The outline of the designs of interest is justified in the General Design Layouts section, and the layouts of interest are classified into two categories, corresponding to extending and contracting devices. The study of the design of extending devices is presented in the Design of Extending Devices section. A similar derivation for the design of contracting devices is reported in the Design of Contracting Devices section. The main design principles derived for extending and contracting devices are summarized in the Summary section. In the Application to Manipulator Design section, the design principles are applied to the design of a bending device in a prototypical scenario. FE simulations to determine two parameters of the bending device in the prototypical scenario are reported in the FE Simulations section. The use of the FE simulations to verify the work is also presented in the FE Simulations section, which leads to the conclusions in the Conclusion section.

## Problem Formulation

The purpose of the devices considered here is to provide a desired motion between two points on the device, which in this case is associated to bending, together with a certain force. In soft robots with fluidic actuation, the motion is achieved by pressurizing a set of chambers in the device to produce the structural deformation. The most common scenario of interest is that where the robot must generate work to produce the motion, overcoming external forces and moments. However, the study presented in this article is completely general, without limitations on the possible designs or on the operational scenario.

The design problem is to select the geometry and structural properties of the soft robot to achieve the desired motion and maximize a specified performance. In this work, the design problem considered is completely general, without predefined design variables. Solving this problem generally requires determining the solution to a nonlinear structural problem with large deformations, for which analytical solutions are not available in general. Thus, an innovative approach is required, as presented in the following sections.

The maximum pressure that a soft robot design can withstand can be very complex to determine, hindering subsequent design optimization. Frequently, however, the pressure limit is primarily dictated by the sealing points in the chambers. In addition, in the common case of medical applications, the maximum pressure can also be limited to guarantee the safety of the patient during a malfunction of the device. The study developed in this work is therefore focused on design optimization for a given maximum pressure.

The performance criteria for the optimization must be related to the purpose of these devices, that is, to provide a bending motion while supporting external forces and moments. Typically, soft robotic manipulators are required to be capable of reaching a specific deflection determined by the desired workspace. The forces and moments they can support at that deflection tend to be their main limitation. In this regard, the optimization objective selected in this work is to maximize the forces and moments that can be supported while achieving a desired deflection and with a given maximum pressure.

## General Design Layouts

The wide diversity of design possibilities makes it difficult to directly address the general design problem and determine the design. It is therefore appropriate to first outline the design space and then use a detailed study to derive the design principles. In this work, a preliminary analysis is first used to bound the design space and to discretize the design options, as described in this section. This enables a subsequent detailed study of the two layouts of interest, which is derived in the following sections. It should be noted that the analysis in this section is general and independent of the desired performance criteria; the study is then particularized in the subsequent sections to the performance criteria selected for this work.

**FIG. 1.**
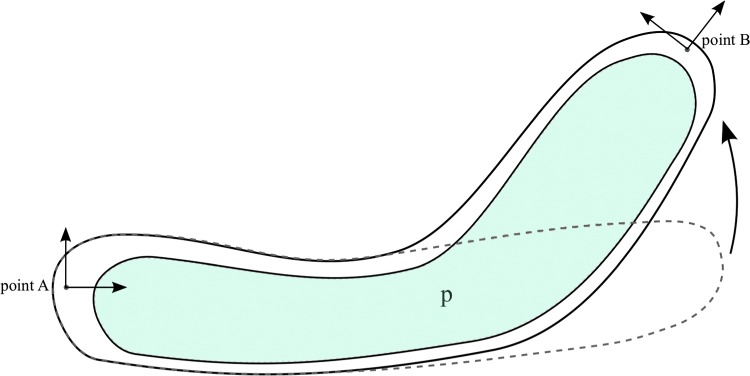
Schematic diagram of a bending device with a completely general design.

Any potential design must consist of a general structure linking the two points of interest, as illustrated in Figure 1. In soft robots with fluidic actuation, the structure is passive, and therefore, the design must contain a set of chambers that can be pressurized to generate the desired motion by deforming the structure. This set of chambers must generally cover the region between the two points of interest in a nearly continuous manner, as otherwise parts of the device would act as structures that simply transmit loads, which is not the focus of this work.

**FIG. 2.**
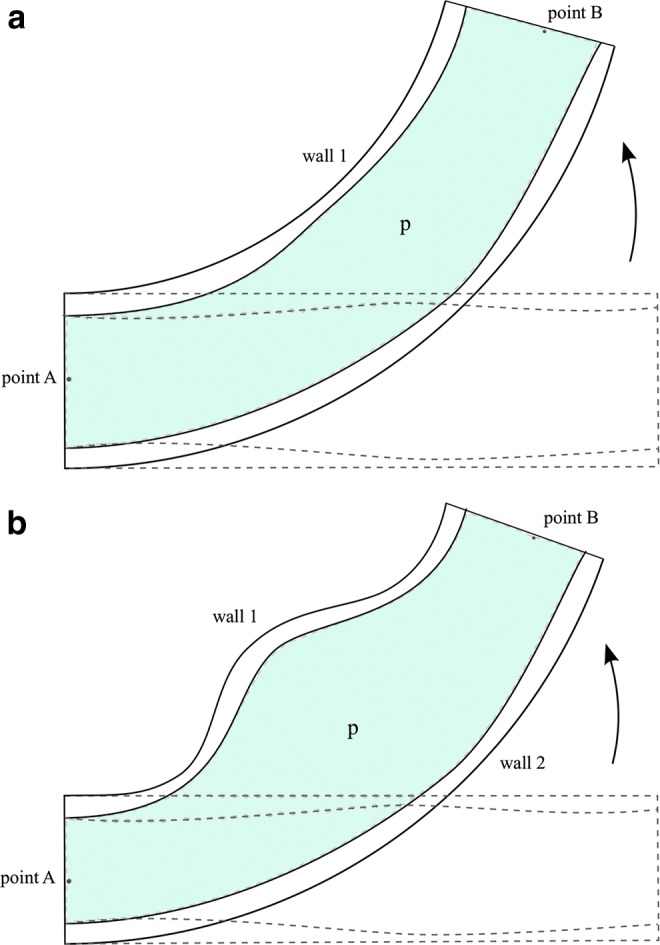
Conceptual illustration of the general layouts corresponding to the two possible types of soft robotic manipulators: **(a)** extending devices and **(b)** contracting devices.

The set of chambers, together with the direction of bending, which is approximately perpendicular to the vector between the two points of interest, define two sides of the device, which can be considered as two walls. Kinematic considerations show that to achieve bending, a differential deformation in the structure at either side of the device is required. This involves either one wall extending more than the other or one wall contracting more than the other. Soft robotic manipulators can therefore generate bending in two elementary ways, and the designs can be classified accordingly, leading to two general categories: extending-type devices and contracting-type devices, as illustrated in Figure 2.

**FIG. 3.**
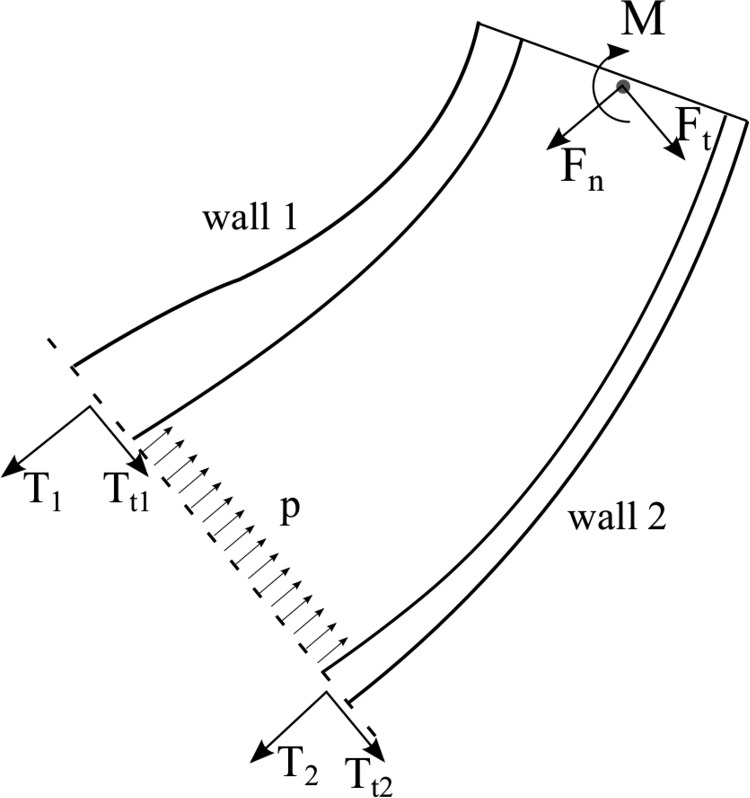
Equilibrium diagram of a general bending device isolated at an arbitrary cross section, exposing the pressure applied by the fluid as well as the structural reactions.

The equilibrium of a system corresponding to the general design isolated at an arbitrary cross section perpendicular to the vector between the two points of interest can then be considered, as shown in Figure 3. This exposes the reaction forces as well as the pressure applied by the fluid. The system equilibrium can thus be used to provide insight into the mechanical behavior and to study the design, and it represents a cornerstone of the analysis presented in this article. Before a detailed study, the equilibrium can first be applied to the two categories of soft robotic manipulators, extending and contracting devices, to outline the design layouts, as described in the following two paragraphs.

Considering the equilibrium in extending devices, this indicates that the pressure in the chambers generally creates tensioning reactions on the structure. The reactions associated to each side of the structure depend on the design. These reactions translate into deformations, with the elongation of each side depending on the stiffness in the longitudinal direction. The differential elongation necessary for bending can therefore be achieved with either an asymmetric pressure loading or an asymmetric longitudinal stiffness. It should be noted that the reactions can also produce lateral expansion, but this generally does not contribute to elongation, rather the opposite, so it is undesirable in extending devices. Thus, the layout of extending devices must consist of an elongated structure that cannot expand radially and has a combination of asymmetric geometry and asymmetric longitudinal stiffness so that one side extends more than the other. The specific combination of geometry and stiffness affects the performance and requires a detailed study, presented in the Design of Extending Devices section.

Considering the equilibrium in contracting devices, this also shows that the pressure generates tensioning reactions. Contraction can therefore not be achieved with a compression of the structure, and instead one side of the structure must either protrude outward or buckle inward. The layout of contracting devices must then consist of a structure with one side that either protrudes or buckles to produce a contraction while the other side maintains the original length, resulting in bending. The principle of operation is similar to that of PAMs, for example, see Ref.,^13^ and some of the analysis can be adapted from there. Still, the equilibrium analysis indicates that both the design geometry and the longitudinal and bending stiffnesses affect the reaction forces, the protrusion geometry, and ultimately the performance, requiring a detailed examination. The study of the design of contracting devices is reported in the Design of Contracting Devices section.

Considering that the extending and contracting devices are the only alternatives to produce bending, the study of these two layouts represents a complete study of the design of soft robotic manipulators with fluidic actuation. Devices combining extension and contraction are also possible, and their design is a combination of the design principles for both types of operation. The design of a device combining both extending and contracting operation is presented in the Summary section.

## Design of Extending Devices

Extending devices achieve bending thanks to a differential extension in their structure when pressurized, which is created by a design asymmetry in terms of geometry and stiffness. The design of extending devices is studied in detail in this section to derive a set of design principles and to determine the design that maximizes the design objective.

Considering that the design objective is to achieve a desired deflection and maximize the force for a given maximum pressure, the study is divided into two parts. First, the study is focused on the design to maximize the forces and moments that can be supported at a given deflection with a constrained pressure, as described in the Equilibrium Approach, Deflection Condition, and Design Derivation sections. Then, the analysis considers the design objective of reaching the desired deflection with a minimum pressure, as presented in the Initial Deflection section. The results of both analyses are combined to extract design principles and determine the most suitable design, summarized in the Complete Design section, while the overall analysis is finally generalized to 3D in the Generalization to 3D section.

### Equilibrium approach

#### Equilibrium formulation

**FIG. 4.**
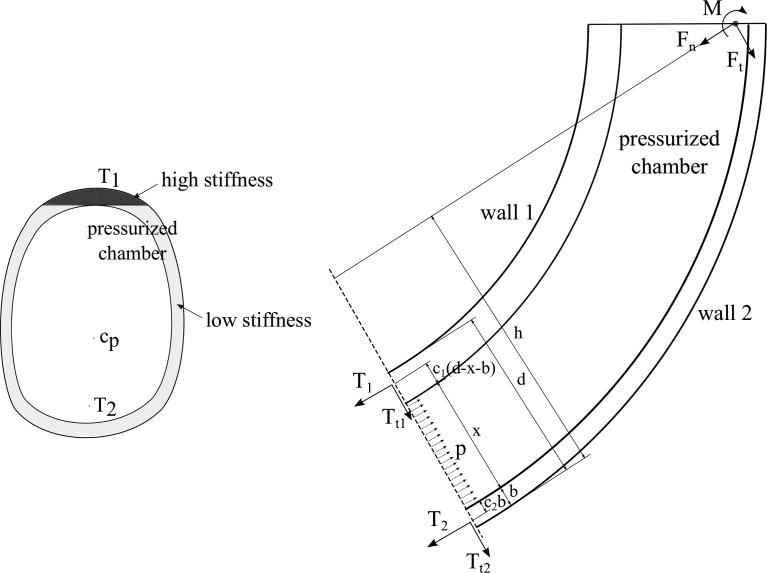
Equilibrium diagram of the extending device isolated at an arbitrary cross section (*right*), exposing the reaction forces, aggregated into *T*_1_ and *T*_2_, and the pressure applied by the fluid. General cross section of a 3D device with variable stiffness (*left*), with the regions in *dark* and *light gray* indicating higher stiffness and lower stiffness, respectively. The approximate lines of application of *T*_1_ and *T*_2_ and the center of pressure *c_p_* are also indicated.

The equilibrium of an extending device isolated at an arbitrary cross section can be considered, as shown in Figure 4 (right), exposing the reactions as well as the pressure applied by the fluid. The equilibrium of moments and forces in the direction perpendicular to the cross section can thus be imposed as

\begin{align*}
& { { T_1 } + { T_2 } = px - { F_n } } \\ &{ { T_1 } \left( { {
c_1 } d + x \left( { 1 - { c_1 } } \right) + b \left( { { c_2 } -
{ c_1 } } \right) } \right)\quad\quad\quad\quad\quad, } \\ &
\quad- p \frac { { { x^2 } } } { 2 } - px { c_2 } b + { F_n }
\left( { h - b \left( { 1 - { c_2 } } \right) } \right) = M \tag {
1 }
\end{align*}

where *d* denotes the total region of the cross section, *x* represents the region of the cross section corresponding to the pressurized fluid, and *b* is the region of the cross section corresponding to wall 2. The external forces are decomposed into two directions, parallel and perpendicular to the cross section. The perpendicular forces are aggregated into a resulting normal force, denoted by *F_n_*, and the parallel forces are aggregated into a resulting tangential force *F_t_*. *M* corresponds to the sum of external moments together with the moment created by *F_t_* with respect to the cross section.

The distributed normal stresses corresponding to wall 1 and wall 2 are aggregated into two equivalent forces, denoted by *T*_1_ and *T*_2_, respectively, whereas the distributed tangential stresses are aggregated into ${T_{t1}}$ and ${T_{t2}}$, respectively. The location of the equivalent line of application of *T*_1_ and *T*_2_ is defined by the nondimensional parameters *c*_1_ and *c*_2_, respectively. The specific equivalent line of application of these two forces may not be constant and can be difficult to determine as it depends on the specific stress distribution, which is determined by a complex structural behavior. However, considering that, in soft robots with fluidic actuation, and particularly in extending devices, the walls are in tension, the equivalent point of application of *T*_1_ and *T*_2_ must be within the respective walls. Thus, the variables *c*_1_ and *c*_2_ are bounded ${c_1} , {c_2} \in \left[ {0 , 1} \right]$. As will be seen in the following, the walls should be thin, and therefore, the stress distribution can generally be considered to be relatively uniform, leading to values of *c*_1_ and *c*_2_ near $1 / 2$. However, the specific point of application does not affect the subsequent derivation and therefore need not be considered further.

The description of the cross section with *d*, *x*, and *b* is convenient, as *d* is generally a parameter determined by constraints from the environment, and then, the design study involves selecting the variables *x* and *b*. It should be noted that the variables *x*, *b*, and *d* are then geometrically bounded. In particular, $x  >  0$, $b  >  0$, $d  >  x  +  b$. Thus, some of the constraints are coupled. It should also be noted that for extending devices to operate, ${F_n}  <  px$.

The device can be subjected to any combination of external forces and moments. The point of application of *F_n_* is determined by the specific external forces in each scenario. The contribution of *F_n_* to the moments equation in Equation (1) depends on the distance between the line of application of *F_n_* and the line of application of *T*_2_. The *F_n_* applied may thus influence the *M* that can be supported and *vice versa*. However, maintaining the contribution of *F_n_* to the moments as a separate force with a certain point of application is desirable as it shows the moments and equivalent moments generated by *F_t_* that can be supported by a design and the effect of *F_n_* on *M*.

#### Equilibrium discussion

Equation (1) indicates that *b* affects the contribution of *F_n_* to *M* through the term ${F_n}b \left( {1 - {c_2}} \right)$, which has an effect on the device's performance. However, this is due to the fact that changes in *b* involve displacing the point of application of *T*_2_. Equivalent alternatives for displacing the point of application of *T*_2_ relative to the point of application of *F_n_* include displacing the entire wall 2 or displacing the entire device. However, any possible offset of the external forces relative to the device to improve performance is considered to be already applied in practice. The problem of interest in terms of design is to maximize performance for a given external loading. In this regard, the effect of varying *b* on ${F_n}b \left( {1 - {c_2}} \right)$ is not relevant from a design perspective as it is equivalent to offsetting the device, and it is therefore disregarded in the design derivation.

The cross section where equilibrium is considered is arbitrary, and therefore, the analysis can be applied to any cross section on the device. This provides insight into the mechanical behavior of the entire device, and therefore, it serves to study the design.

The equilibrium of forces also shows that external forces parallel to the cross section must be supported at the boundary where the device is isolated. Considering the definition of fluid, the direction of the pressure force is always normal to the boundary. Thus, the lateral forces must be supported by the structure in any design, particularly by ${T_{t1}}$ and ${T_{t2}}$. The contribution of this shear stress to the deflection, however, is considered to be relatively small, following the standard study of structures. In this regard, the equilibrium in the direction parallel to the cross section is not considered further.

The system of equations (Equation 1) provides the reactions *T*_1_ and *T*_2_ for any *M* and *p* given a design. These solutions, however, correspond to different structural deformations and therefore different displacements. Thus, the equilibrium alone cannot be used to determine the design to maximize *M*, as a combination of *T*_1_ and *T*_2_ to increase *M* always exists, but it may correspond to an undesirable deflection. To study the design for a given deflection of interest, a condition imposing a desired deflection to be maintained is required.

### Deflection condition

The purpose of the deflection condition is to define the relation between *T*_1_ and *T*_2_ that must be satisfied for a desired deflection to remain constant. In particular, the deflection must remain constant despite variations in the external forces and moments, as well as pressure applied.

Deflection depends on the differential wall extension. Thus, deflection can be maintained even at different pressures provided that both walls extend. The deflection condition can therefore not be determined from a specified extension value at each wall, but rather must be derived from a ratio between the extensions of both walls.

To attain a desired deflection, even without external forces or moments, a certain extension at each wall is necessary, which corresponds to the initial extension of the walls. Once the initial deflection is achieved, it can be maintained even for variable external forces and moments by compensating with pressure. More specifically, deflection can be maintained at variable values of wall extension provided that any increase in length in a wall is accompanied by a certain increase in length at the other wall. A condition to maintain a deflection can therefore be obtained by imposing the increase in length at both walls to be related through a certain ratio *R* as

\begin{align*}
{ \delta _1} = R{ \delta _2} , \tag{2}
\end{align*}

where ${ \delta _i}$ denotes the increase in length in wall *i* with respect to the length necessary to attain the initial deflection. The value of the ratio *R* is generally close to 1, but it can depend on the desired deflection. However, the derivation in this work does not require the exact value of *R*, and it is therefore not specified. It should be noted that any variation in extension must be associated with a variation both in external forces and moments and in pressure.

The extension in a wall depends on both the stress applied and the wall stiffness. In addition, the initial extension required in each wall to reach the initial deflection involves a certain initial tension ${T_{i0}}$ for $i = 1 , 2$. In this regard, the deflection condition cannot simply impose a relation between *T*_1_ and *T*_2_, but it must include the stiffnesses of the walls as well as the initial tension of the walls. The increase in extension ${ \delta _i}$ in a wall *i* can be related to the increase in tension in that wall ${T_i} - {T_{i0}}$ through a variable stiffness *s_i_* as

\begin{align*}
{T_i} - {T_{i0}} = {s_i}{ \delta _i}. \tag{3}
\end{align*}

The value of *s_i_* can be difficult to determine, and it is not necessarily constant. In general, *s_i_* can depend on the material, the design, and the deformation. However, the specific *s_i_* is not calculated here since it is not necessary for the derivation.

Substituting the relation between extension and tension (Equation 3) into Equation (2), the condition that must be satisfied for a deflection to be maintained is obtained as

\begin{align*}
 { T_2 } = { \frac { { T_1 } { s_2 } }  { R { s_1 } } } + { T_ { 20 } } - { \frac { { T_ { 10 } } { s_2 } }  { R { s_1 } } } . \tag { 4 } 
\end{align*}

The deflection condition is thus expressed as a relation between *T*_1_ and *T*_2_, as well as a set of parameters.

This condition (Equation 4) is applicable to any scenario with any desired deflection and combination of external forces and moments. The two terms on the right depend on the conditions to achieve initial deflection, and thus, the desired deflection in each scenario is imposed by these terms. These two terms are constant and are analyzed in the Initial Deflection section. The value of *R* may also vary to some extent for some of these different scenarios, although in some instances, the value of *R* can be equal for different deflections. Still, all these parameters are specified for a given scenario. Thus, Equation (4) defines the relation between *T*_1_ and *T*_2_ that guarantees the deflection to be maintained in any scenario.

Interestingly, in the case of infinite stiffness at wall 1, the deflection condition (Equation 4) simply imposes *T*_2_ to be constant. This is a typical situation as will be seen in the following, where designs with infinite wall 1 stiffness are particularly relevant. However, a constant *T*_2_ is not a valid condition to maintain deflection in general, since, in extending devices, wall 2 may need to extend to a certain degree as pressure increases to compensate the extension in wall 1.

### Design derivation

The equilibrium and the deflection condition can be combined to analyze the design problem and derive a set of design principles, as described in the following sections.

#### Preliminary qualitative considerations

The equilibrium analysis, illustrated in Figure 4 (right), indicates that the moment at the cross section necessary to support external moments as well as the equivalent moments generated by external forces are created between the pressure and the reactions. In particular, since pressure can only act in one direction, and the structure generally acts in the opposite direction, the moment is created between the pressure pushing and the structure pulling.

The main challenge is supporting forces and moments that tend to reduce the deflection, that is, forces and moments that contribute as positive values of *M*. Opposite forces and moments increase the deflection and supporting them is thus trivial.

The pressure is always acting between the two walls in tension. Thus, the moment must be created between *T*_1_ pulling and *p* pushing. *T*_2_, however, opposes to this moment and is therefore undesirable in general. The only purpose of wall 2 is to contain the pressurized fluid.

This qualitative analysis indicates that maximum *T*_1_ and minimum *T*_2_ are desirable. This could lead to the impression that concentrating the pressure application near wall 1, for example, using thick or even hollow structure in wall 2, maximizes performance, as it maximizes *T*_1_ and minimizes *T*_2_. However, this arrangement also promotes an undesirable deflection. In the extreme case, a design with ${T_2} = 0$ and thus ${T_1} = px$ would be possible, but it would yield zero or negative deflection, which is undesirable as deflection must be maintained. Conversely, concentrating the pressure application close to wall 2 would minimize the increase in *T*_1_, and thus, the reduction in deflection when pressure is increased, enabling *p* to compensate generate the majority of the moment without loss of deflection. However, this also results in low *T*_1_ and thus low forces and moments that can be supported. The analysis combining the equilibrium (Equation 1) and deflection condition (Equation 4) is derived in the following section to resolve these design questions.

#### Detailed analysis and derivation

Imposing the condition requiring a deflection to be maintained (Equation 4) into the equilibrium of forces in (Equation 1) yields

\begin{align*}
 { T_1 } = { \frac { px - { F_n } - \kappa }  { 1 + { \frac { { s_2 } }  { R { s_1 } } } } } , \tag { 5 } 
\end{align*}

where $\kappa = {T_{20}} - {T_{10}}{s_2} / R{s_1}$, which corresponds to the initial deflection conditions. Substituting Equation (5) into the equilibrium of moments in Equation (1), the *M* can be supported for a given design and a certain deflection is obtained as

\begin{align*}
{{px - {F_n} - \kappa } \over {1 + {{{s_2}} \over {R{s_1}}}}}
\left[ {{c_1}d + x \left( {1 - {c_1}} \right) + b \left( {{c_2} -
{c_1}} \right) } \right] \\ {- p{{{x^2}} \over 2} - px{c_2}b +
{F_n} \left( {h - b \left( {1 - {c_2}} \right) } \right)} = M.
 \tag{6}
\end{align*}

It should be noted that, as previously mentioned, ${T_1}  >  0$ for operation to be possible since otherwise the structure would be in compression, and the device would not act as an extending device but rather as a passive structure. Thus, from Equation (5), this implies a bound $px - {F_n} - \kappa  >  0$.

Equation (6) enables determining the design to maximize the desired performance, which in this case involves maximizing *M*. Equation (6) is applicable to any deflection, and therefore, it can be used to address the design problem in any scenario. It should be noted that the effect of the terms corresponding to initial deflection, aggregated in $\kappa$, is studied separately in the Initial Deflection section.

The design principles can be extracted by considering the contribution of the design variables to *M* in Equation (6). The stiffnesses *s*_1_ and *s*_2_ appear only as a ratio ${s_2} / {s_1}$. The ratio only contributes to the denominator of a term that should be maximized for the case of interest $px - {F_n} - \kappa  >  0$, and therefore, ${s_2} / {s_1}$ should be minimized. As previously mentioned, the values of *s*_1_ and *s*_2_ may depend on the design as well as the material. However, in soft robotic devices, the material can generally be chosen to provide any desired stiffness, particularly including low values. In this regard, the material can be used to select *s*_1_ and *s*_2_, compensating for any variation in stiffness associated to the geometry. Thus, the minimization of ${s_2} / {s_1}$ is considered to be attainable with the material choice, independent of the rest of the design.

The variables *s*_1_ and *s*_2_ represent the overall stiffness of a wall, but the local stiffness within the wall needs not be constant. The specific stiffness distribution affects the line of application of *T*_1_ and *T*_2_ and therefore can be used to modify *c*_1_ and *c*_2_. The line of application is determined by the location where the moment generated by the distributed stress within a wall is equal to that created by *T*_1_ or *T*_2_. For a given wall in extension, corresponding to a deflection, the normal stress within the wall can be considered to be strongly dependent on the local stiffness, especially if the stiffness distribution over the cross section presents significant differences. Thus, the wall layers with markedly higher stiffness generally involve higher local stress, and the line of application of the equivalent force can be considered to tend to these layers.

The stiffness distribution can therefore be used to modify *c*_1_ and *c*_2_. However, it should only be used for *c*_1_. Considering that *s*_2_ should be minimized, and that low stiffness is difficult to attain, any stiffness variation typically involves an increase in *s*_2_, reducing the performance. Instead, a high *s*_1_ can generally be maintained since local stiffness can typically be increased to compensate local reductions. Equation (6) indicates that a high *c*_1_ is desirable, and therefore, wall 1 should have a high stiffness in the outer layers and lower stiffness in the inner layers. Still, this is only relevant in designs where wall thickness is substantial, which are typically not the designs of interest, as shown subsequently.

For an ${s_2} / R{s_1}$ that is minimized, $\kappa$ is typically negligible, as can be seen from the analysis in the following section. The derivation of the rest of design principles can then be divided in two cases for clarity of exposition.

#### Case with *F_n_* = 0 and negligible κ

A case with ${F_n} = 0$ and $\kappa$ negligible can be considered first as it represents a common scenario of interest where the robotic manipulator must support external forces in the direction of bending, as in a nearly horizontal robot segment supporting and moving a payload against gravity, or a nearly horizontal segment moving a set of additional segments stacked serially at its distal end, which generates an external lateral force and moment. In addition, the case with ${F_n} = 0$ and *k* negligible provides a first intuitive understanding of the design principles. In this case, and for ${s_2} / R{s_1}$ negligible relative to 1, each of the variables $b , x , d$ only affects one or a small number of terms in Equation (6) and thus can be easily determined. In addition, *p* can be factorized, so the desired value of these variables is independent of pressure.

The variable *b* affects three terms, the combination of which always reduces *M* since ${s_2} / R{s_1}  >  = 0$, and *x*, *c*_1_, and *c*_2_ are nonnegative. Hence, *b* should be minimized, which can be written as $b = 0$. Then, for $b = 0$, the value of *x* to maximize *M* depends on *c*_1_, ${s_2} / R{s_1}$, and *d*. If $1 - 2{c_1} - s2 / Rs1  >  0$, then *x* should be maximized and therefore $x = d$. If $1 - 2{c_1} -  s2 / Rs1  <  0$, then the value of *x* to maximize *M* is

\begin{align*}
x = { \frac { 2 { c_1 } d }  { 2 { c_1 } + s2 / Rs1 - 1 } } . \tag { 7 } 
\end{align*}

Considering that ${s_2} / R{s_1}$ should be minimized, this implies $x = d$. Thus, in a design where ${s_2} / R{s_1}$ is minimal, the most suitable cross section is $x = d$ regardless of the value of the parameters *c*_1_ and *c*_2_. The design of the cross section with maximal *x* and minimal *b*, so that the cross-sectional region corresponding to the pressurized fluid is maximal, in designs were ${s_2} / R{s_1}$ is minimized, represents another relevant design principle. It should be noted that in some practical cases it may not be possible to minimize ${s_2} / R{s_1}$ due to manufacturing or material constraints. In these cases, *x* is determined by Equation (7), which may be lower than $x = d$.

Finally, the parameter *d* is determined by the practical application, but Equation (6) highlights that increasing *d* results in higher force. Thus, *d* should be maximized to occupy all available room in each scenario.

#### Case with *F_n_*, κ ≠ 0

Considering a general scenario including *F_n_* and $\kappa$, a similar analysis can be applied to determine the design principles. In this case, the contribution of ${F_n}b \left( {1 - {c_2}} \right)$ to *M* in Equation (6) should be disregarded, as discussed in the Equilibrium Approach section. Then, for a ${s_2} / R{s_1}$ negligible relative to 1, the variables *x* and *b* can be analyzed in conjunction. The analysis is divided in two further cases depending on the sign of ${F_n} + \kappa$.

For ${F_n} + \kappa  <  0$, the terms in Equation (6) containing either of these variables can be aggregated into three groups: terms containing *xb*, terms containing sums of *x* and *b*, and terms containing only *x*, as

\begin{align*}
& { { \tau _1 } = - pxb { c_1 } } \\ & { { \tau _2 } = \left( { -
{ F_n } - \kappa } \right) \left[ { { c_1 } d + x \left( { 1 - {
c_1 } } \right) + b \left( { { c_2 } - { c_1 } } \right) }
\right]. }\\ & { { \tau _3 } = px \frac { { x \left( { 1 - 2 { c_1
} } \right) + 2 { c_1 } d } }  { 2 } } \tag { 8 }
\end{align*}

The terms corresponding to ${ \tau _1}$ reduce *M* and should therefore be minimized, which entails that either *x* or *b* should be minimized. The term corresponding to ${ \tau _2}$ should be maximized, which, considering that $b + x  <  d$, implies that combinations of *x* and *b* that yield $b + x = d$ are desirable. In particular, if a trade-off between *x* and *b* is possible, combinations with higher values of *x* are preferable since the contribution of *x* to ${ \tau _2}$ is higher. Finally, the terms corresponding to ${ \tau _3}$ contribute to *M* and should therefore be maximized.

The value of *x* to maximize ${ \tau _3}$ deserves consideration as the relation between ${ \tau _3}$ and *x* is parabolic. If ${c_1}  >  1 / 2$, then ${ \tau _3}$ is maximized with the specific value of *x*

\begin{align*}
 { x_m } = - { \frac { { c_1 } d }  { 1 - 2 { c_1 } } } , \tag { 9 } 
\end{align*}

which is always ${x_m}  >  = d$. Considering the constraint $x  <  d$, the value of *x* should then be $x = d$. If ${c_1}  <  1 / 2$, then ${ \tau _3}$ as a function of *x* is a parabola that tends to infinity and intersects the *x* axis at 0 and at a negative value. Hence, *x* should also be maximized. Thus, for any *c*_1_ within the possible values, if ${s_2} / R{s_1}$ can be minimized as previously discussed, then the *x* to maximize *T*_3_ should be $x = d$.

The desirable values of *x* and *b* can thus be determined. ${ \tau _1}$ requires either *x* or *b* to be minimized, ${ \tau _2}$ indicates that a trade-off between *x* and *b* be achieved, prioritizing *x*, and ${ \tau _3}$ requires *x* to be maximized. Hence, *b* should be minimized, which can be expressed as $b = 0$, and *x* should be maximized, yielding $x = d$.

For ${F_n} + \kappa  >  0$, a similar derivation can be used. Defining a change of variable $y = px - {F_n} - \kappa$, the terms in Equation (6) containing either *y* or *b* can be aggregated into three groups: terms containing *yb*, terms containing only *b*, and terms containing only *y*, as

\begin{align*}
&{ { \tau _1 } { \rm { \prime } } = - y { c_1 } b } \\ & { { \tau
_2 } { \rm { \prime } } = - \left( { { F_n } + \kappa } \right) {
c_2 } b\quad\quad\quad\quad\quad\quad\quad\quad\quad\quad. } \\ &
{ { \tau _3 } { \rm { \prime } } = y { c_1 } d + \frac { { { y^2 }
} } { p } \left( { \frac { { 1 - 2 { c_1 } } } { 2 } } \right) -
\frac { { y \left( { { F_n } + \kappa } \right) { c_1 } } }  { p }
} \tag { 10 }
\end{align*}

The terms corresponding to both ${ \tau _1} \prime$ and ${ \tau _2} \prime$ reduce *M* and thus should be minimized. Instead, the terms corresponding to ${ \tau _3} \prime$ increase *M* and should be maximized.

As in the previous case, the maximization of ${ \tau _3} \prime$ requires some consideration. If ${c_1}  <  1 / 2$, then the relation between *y* and ${ \tau _3} \prime$ is a positive parabola that intersects the *y* axis at 0 and at a negative value, since ${F_n} + \kappa  <  px$. Thus, *y* should be maximized. If ${c_1}  >  1 / 2$, then ${ \tau _3} \prime$ as a function of *y* is a negative parabola that is maximized at

\begin{align*}
 { y_m } = - { \frac { p { c_1 } d - \left( { { F_n } + \kappa } \right) { c_1 } }  { 1 - { c_1 } } } , \tag { 11 } 
\end{align*}

which is always ${y_m}  >  pd - {F_n} - \kappa$. The value of *y*, however, is bounded $0  <  y  <  pd - {F_n} - \kappa$ since $px  >  {F_n} + \kappa$ and $x  <  d$. Thus, for ${c_1}  >  1 / 2$, *y* should also be maximized.

The desirable values of *y* and *b* to maximize ${ \tau _3} \prime$ and minimize ${ \tau _1} \prime , { \tau _2} \prime$ are maximum *y* and minimum *b*. Reversing the change of variable $y = px - {F_n} - \kappa$, this implies $b = 0$ and $x = d$.

The design principles for all admissible values of ${F_n} + \kappa$ are therefore equal to those in the case where ${F_n} = 0$. Hence, these constitute general principles to maximize the *M* that can be supported at a given deflection.

#### Final derivation considerations

This analysis was derived considering the equilibrium in an arbitrary cross section, and therefore, it is applicable to any cross section on the device. In addition, it also applies to any deflection, pressure, and combination of external forces and moments. Thus, the design principles can generally be used to determine the design of a device to maximize the forces and moments that can be supported.

For a given design and deflection, both the reactions at the cross section and *p* vary with any external forces and moments applied to create the moment that maintains equilibrium. Specifically, for an increase in *M*, both *p* and *T*_1_ must increase. If *s*_1_ is not negligible, then the increase in *T*_1_ is accompanied by an increase in *T*_2_ that maintains deflection, with a ratio that depends on ${s_2} / R{s_1}$. However, as discussed in the previous subsections (Detailed Analysis and Derivation; Case with *F_n_* = 0 and negligible κ; and Case with *F_n_*, κ≠0), ${s_2} / R{s_1}$ should always be minimized, and therefore, the increase in *T*_2_ is generally low.

The moment created by the external forces can vary in different cross sections. However, the design to maximize performance remains equal in all cross sections, regardless of the equivalent moments, as argued in the previous paragraphs. The variable moment in different cross sections can result in uneven deformation along the device but that simply implies a small variation in *R*, which does not affect the derivation. Thus, a constant cross-sectional design throughout the device, with a design determined by the design principles derived in previous paragraphs, is the most suitable design solution in general.

#### Derivation discussion

It should be noted that, in designs determined by the design principles derived here, bending is mainly achieved with a differential stiffness in the two sides of the structure, rather than an asymmetric geometry. The values of *T*_1_ and *T*_2_ can therefore be equal, but the different longitudinal stiffness in both walls produces the deflection. In addition, when external forces and moments are supported, *T*_2_ can be lower than *T*_1_, but the deflection can be maintained thanks to the different stiffness in both walls.

The designs principles derived here are valid for *T*_1_ and *T*_2_ with a line of application anywhere within the wall thickness. Thus, even singular designs with a hollow wall structures to create separation are considered, but these are undesirable according to the design principles, which is a consequence of the fact that maximizing the cross-sectional region corresponding to the pressurized fluid is always desirable. In this regard, the results of the design analysis are general in terms of maximizing the force of the device at a given deflection.

The analysis indicates that the forces and moments that can be supported depend on the maximum pressure. Thus, if the pressure limit was infinite, the device would be capable of supporting practically any forces and moments, which illustrates the potential of soft robots with fluidic actuation. Still, elongation of the device would occur for finite *s*_1_, complicating the practical implementation.

It should also be noted that the design principles only require the ratio ${s_2} / {s_1}$ to be minimized, but the absolute value is not imposed. This could lead to the false impression that the absolute stiffness is not relevant to the device performance. However, the absolute stiffness affects the pressure required to reach the desired deflection, as described subsequently.

### Initial deflection

A similar approach as that described in previous subsections (Equilibrium Approach; Deflection Condition; and Design Derivation) is applied here to study the most suitable design to attain a desired deflection with minimum pressure. The same equilibrium of the device isolated at an arbitrary cross section can be considered, as illustrated in Figure 4 (right). This provides the reactions for a given cross-sectional design.

Deflection is achieved with a differential extension of the walls. This can be attained with a difference between *T*_1_ and *T*_2_, a difference in stiffness of the walls, or a combination. The absolute extension in a wall *i*, denoted by ${{\Delta}_i}$, can be related to the tension using a similar expression as Equation (3), but here in absolute terms

\begin{align*}
{T_i} = {s_i}{{\Delta}_i}. \tag{12}
\end{align*}

As described in the Deflection Condition section, *s_i_* can be difficult to determine, but the specific value is not necessary for the derivation and is therefore not considered further. The use of Equation (12) is advantageous as it elucidates the two methods to achieve deflection.

To attain the desired deflection with minimum pressure, it is necessary to facilitate achieving the desired difference between ${{\Delta}_1}$ and ${{\Delta}_2}$. Using Equation (12), the desired differential extension of the walls can be expressed as

\begin{align*}
{{\Delta}_2} - {{\Delta}_1} = {T_2} / {s_2} - {T_1} / {s_1}. \tag{13}
\end{align*}

Thus, in terms of stiffness, the difference between *s*_1_ and *s*_2_ should be maximized. It should be noted that maximizing the difference between *s*_1_ and *s*_2_ facilitates attaining the desired deflection regardless of the tensions in the walls. In this regard, it represents a general principle in terms of attaining the desired deflection at minimum pressure.

In terms of tensions, Equation (13) elucidates that difference between *T*_1_ and *T*_2_ should also be maximized for a given *p*. Since *s*_1_ should be maximized and *s*_2_ minimized, the determining factor in Equation (13) to maximize deflection is *T*_2_, which should be maximized. Considering the equilibrium (Equation 1), and after some manipulation, it can be seen that the tensions depend on the cross-sectional design as

\begin{align*}
& { { T_1 } = { \frac { p \frac { { { x^2 } } }  { 2 } + px { c_2
} b + M + { F_n } \left( { h - { c_2 } b } \right) }  { x \left( {
1 - { c_1 } + d { c_1 } + b \left( { { c_2 } - { c_1 } } \right) }
\right) } } } \\ & { { T_2 } = { \frac { \frac { { p { x^2 } } } {
2 } \left( { 1 - { c_1 } } \right) + px { c_1 } \left( { d - b }
\right) - M + { F_n } \left( { h - b { c_2 } } \right) }  { x
\left( { 1 - { c_1 } } \right) + d { c_1 } + b \left( { { c_2 } -
{ c_1 } } \right) } } }. \tag { 14 }
\end{align*}

As discussed in the Equilibrium Approach section, the contribution of the term ${F_n}b \left( {1 - {c_1}} \right)$ is disregarded since it is equivalent to offsetting the device. Then, from Equation (14), it can be seen that for $M  >  0$, which are the equivalent external moments of interest as previously discussed, reducing *b* to increase *x* is always desirable since $\partial {T_2} / \partial b  <  0$ and $\partial {T_2} / \partial x  >  0$. Thus, to maximize *T*_2_ and therefore deflection, *b* should be minimized and *x* should be maximized, which can be written as $b = 0$ and $x = d$. For $x = d$, Equation (14) also elucidates that a maximum *d* is desirable, hence *d* should be selected to occupy all space available.

Interestingly, the performance in terms of initial deflection depends on the absolute stiffness of the walls, as elucidated in Equation (13). Hence, for a given difference between *s*_1_ and *s*_2_ that cannot be increased, the absolute stiffness should be minimized to achieve deflection at minimum pressure.

The analysis in this section therefore indicates that the design principles to attain a desired deflection with minimum pressure are maximum *s*_1_, minimum *s*_2_, $b = 0$, $x = d$, maximum *d*, and minimum absolute stiffness when possible. It should be noted that these equalities in practice denote that the variables should tend to the desired values, that is, minimum wall thickness and maximum region corresponding to the pressurized fluid. In the optimal design $x = d$, Equation (14) indicates that *T*_2_ increases with *d*, which should therefore be maximized to occupy all available room in each scenario. The derivation of these results is independent of the desired deflection or the pressure, and therefore, they represent general principles.

### Complete design

The designs to maximize the external forces and moments that can be supported at a given deflection and maximum pressure, and to achieve a deflection at minimum pressure, were elucidated in the two previous sections. The design objective in this work involves attaining a desired deflection and maximizing the forces and moments that can be supported with a given maximum pressure, which couples both analyses.

Fortunately, there is an agreement in the design principles to achieve both objectives, as summarized subsequently. The ratio ${s_1} / {s_2}$ should be maximized in both cases, which can be attained, for example, with a pleated structure in wall 2. Then, for a high ${s_1} / {s_2}$, *x* in both cases should be maximized, *d* should be maximized, and *b* should be minimized. The only difference is that the absolute values of *s*_1_ and *s*_2_ are not relevant in terms of maximizing force at a given deflection and maximum pressure, but they are relevant to attain the desired deflection at minimum pressure. Thus, absolute stiffness should generally be minimized.

This applies to any cross section on the device and to any deflection and pressure value. Thus, these design principles summarized in the previous paragraph can be used to determine the most suitable design. Since the design principles are independent of the maximum pressure and the deflection, the most suitable design is relatively independent of the desired application.

### Generalization to 3D

The study up to this point considered a planar scenario. The generalization to 3D is presented in this section. The analysis in 3D is mostly analogous; it involves considering the equilibrium of a device isolated in a cross section, aggregating the distributed reactions onto two tensioning force variables, distilling a condition to maintain deflection and combining them to determine the design. However, the generalization of elements such as the aggregation of forces and deflection condition requires a careful examination.

In the 3D scenario, the soft robotic manipulator is considered to bend in a desired plane. External forces are considered to act in the plane of bending, as it represents the most relevant case for the design study. This scenario lends itself to the analysis of symmetric designs, but this symmetry is not used in the derivation to maintain generality of the study. The study can then be directly extrapolated to the design of devices capable of supporting out of plane forces.

The 3D device isolated in an arbitrary cross section can be considered, as in 2D. Here, the force associated to the pressure is *pA*, where *A* is the area of the cross section corresponding to the chamber, and *p* is pressure as before. The force *pA* is applied at the center of pressures, which depends on the chamber geometry.

#### Aggregation of forces *T*_1_ and *T*_2_

The distributed normal stresses at the cross section can also be aggregated into two forces *T*_1_ and *T*_2_ as in the planar case. However, the specific division of the cross section into two regions, the stresses of which correspond to *T*_1_ and *T*_2_, affects the analysis and therefore must be considered. The moment at the cross section that produces bending and supports external moments and equivalent moments generated by external forces is created between the pressure and distributed reaction stresses at one side of the structure, with the reactions at the other side opposing to it. Thus, a suitable dividing line is that passing through the center of pressures and perpendicular to the bending plane, as it yields a *T*_1_ aggregating all distributed stresses that contribute to the moment and a *T*_2_ aggregating all stresses that oppose to it, as in the planar scenario.

A dividing line passing through the center of pressures implies that the relative location of this line can vary with the cross-sectional design. However, this is desirable, as the cross-sectional stresses that contribute to the moment also depend on the design. Thus, the dividing line proposed here ensures that the stresses are appropriately aggregated since the stresses associated to each force always share a common objective in terms of contribution to the device performance.

It should be noted that, as in the planar case, the equivalent line of application of *T*_1_ and *T*_2_ can be assumed to be within the region of the cross section they correspond to. Indeed, considering that extending devices achieve deflection thanks to a differential extension of the walls and that this is produced with a pressurized fluid, it can generally be assumed that the normal stresses at the cross section are predominantly tensioning stresses, and therefore, *T*_1_ and *T*_2_ are applied within the cross section.

#### Effect of stiffness distribution on *T*_1_ and *T*_2_

The specific line of application of *T*_1_ or *T*_2_ is affected by the stiffness distribution in the region they correspond to, as illustrated in Figure 4 (left). As in the planar case, the stiffness in a region needs not be constant, and specific stiffness distributions can be used to displace *T*_1_ and *T*_2_. *T*_1_ and *T*_2_ are applied at the point where the moment they create is equivalent that was generated by the normal stress in their corresponding region. In designs with a constant cross section and at a certain deflection, the local stress in the cross section can be considered to be higher at the subregions with markedly higher stiffness, particularly when the variations in the stiffness distribution are significant. Thus, the line of application of *T*_1_ and *T*_2_ can be considered to tend to the location of higher stiffness within their regions.

As in the planar case, the desired stiffness is considered to be selectable with the material choice, compensating for any effects from the design geometry. Thus, a typical configuration of interest with *T*_1_ applied at an edge of the cross section can be attained with a high-stiffness material in the desired subregion and a low-stiffness material over the rest of the cross section, as shown in Figure 4 (left). In this case, the line of application of *T*_1_ can be considered to be relatively independent of the cross-sectional geometry.

#### Generalization of deflection condition to 3D

The condition to maintain deflection can also be generalized to 3D. To maintain deflection, the overall normal strain distribution in the cross section should be approximately preserved, which implies that any increase in extension should be relatively homogeneous over the cross section. Considering that the stiffnesses at the cross-sectional regions corresponding to *T*_1_ and *T*_2_ can be anticipated to be markedly different, a stress distribution with two distinct values corresponding to two regions in terms of stiffness can be expected.

The specific relation between *T*_1_ and *T*_2_ to maintain deflection is difficult to determine, as these average values may correspond to different stress and strain distributions. However, a ratio between *T*_1_ and *T*_2_ that guarantees that the deflection maintained must always exist. Indeed, an increase in *M* while *p* and the external forces remain constant results in a decrease in deflection, whereas an increase in *p* while all external forces and moments are constant leads to an increase in deflection. Thus, a configuration where deflection is maintained exists, and this corresponds to a certain ratio between *T*_1_ and *T*_2_. In particular, following a similar structure as in 2D, at each configuration of equilibrium in each cross section, a relation of the type

\begin{align*}
 { T_2 } = { \frac { { T_1 } { S_2 } }  { R { S_1 } } } + { T_ { 20 } } - { \frac { { T_ { 10 } } { S_2 } }  { R { S_1 } } } \tag { 15 } 
\end{align*}

exists, which guarantees that the deflection is maintained with a certain value of *R*. It should be noted that the variables *S*_1_ and *S*_2_ denote the longitudinal stiffnesses of the cross-sectional regions corresponding to *T*_1_ and *T*_2_, respectively, and are analogous to *s*_1_ and *s*_2_ in 2D.

The specific value of *R* can be difficult to determine and may depend on the cross section. In general, considering the discussion in the previous paragraph, it can be bounded to be positive. Provided that it is positive, the specific value of *R* is not relevant to the design derivation in general, as in the planar case, and it is therefore not considered further.

It should be noted that that the existence of the condition (Equation 15) with a certain *R* is independent of the deformation distribution over the cross section. In some cross sections, it can occur that maintaining the deflection with different external forces and moments leads to a somewhat different strain distribution, resulting in a variation in the bending mode of the overall device. However, this only implies a somewhat different *R* in the cross sections, but the overall deflection is maintained. In addition, *R* remains positive in general, which is the main requisite for the derivation of the design principles.

#### Generalization of design derivation to 3D

With these concepts generalized to 3D, the equilibrium of the device isolated at an arbitrary cross section can also be considered in 3D. As in the planar case, the equilibrium indicates that *T*_1_ and *p* generate the moment, and are desirable, whereas *T*_2_ opposes to it. However, for a deflection to be maintained, relation (Equation 15) between *T*_1_ and *T*_2_ must be satisfied. The equilibrium of forces

\begin{align*}
{T_1} + {T_2} = \mathop \int       \int \limits_A { \rm{ }}p{ \rm{ \;}}dA - {F_n} \tag{16}
\end{align*}

can therefore be combined with Equation (15) and substituted into the equilibrium of moments, yielding

\begin{align*}
& - \left( {{F_n} + K} \right) {D \over {1 + {S_2} / R{S_1}}} +
\mathop \int       \int \limits_A { \rm{ }}{{pD} \over {1 + {S_2}
/ R{S_1}}} \\ & - p \chi { \rm{ \;}}dA + {F_n}H = M ,
 \tag{17}
\end{align*}

where *D* is the distance between the line of application of *T*_1_ and *T*_2_, $\chi$ is the distance in the direction of bending between a point in the cross section and the line of application of *T*_2_, generalizing *x* in 2D, *K* is a constant associated to the initial deflection of the device, which generalizes $\kappa$ and is also typically low and positive, *H* is the distance between the line of application of *F_n_* and *T*_2_, generalizing $h - b \left( {1 - {c_2}} \right)$, and the rest of variables are a direct generalization of those in 2D. Both *D* and $\chi$ depend on the design geometry and stiffness distribution. However, $\chi$ does not depend on the line of application of *T*_1_ and therefore is not affected by the stiffness in the region corresponding to *T*_1_. The value of *H* can also vary with the design, but, as in the planar case, this variation is disregarded since it is equivalent to offsetting the device. Equation (17) is equivalent to Equation (6) and can be used to derive the design principles in 3D.

#### Case with *F_n_* + *K* = 0

Considering a case with ${F_n} + K = 0$ first, Equation (17) indicates that ${S_2} / R{S_1}$ should be minimized. Thus, maximal *S*_1_ and minimal *S*_2_ are desirable. As previously discussed, the overall stiffness in a region, *S*_1_ or *S*_2_, can be composed of different stiffnesses in different subregions, which can be used to displace the line of application of *T*_1_ and *T*_2_ toward the subregions of higher stiffness. Equation (17) indicates that *D* should be maximized while maintaining the values of $\chi$, that is, by displacing the line of application of *T*_1_. Hence, the stiffness distribution in the region corresponding to *T*_1_ should be analogous to that in 2D and consists of a high-stiffness subregion near the edge in the direction of bending and a lower stiffness over the rest, as previously introduced and illustrated in Figure 4 (left). This preserves a minimal ${S_2} / R{S_1}$ and maintains *T*_1_ applied near the edge despite variations in the cross-sectional geometry.

The integrand in Equation (17) can then be considered to be always positive. Its local value is the distance between *T*_1_ and a differential element of chamber area, $D - \chi$, which is not affected by variations in the line of application of *T*_2_. Hence, the integrand is relatively independent of design geometry since the line of application of *T*_1_ is relatively constant. Then, the area of the integral in Equation (17) should be maximized to maximize *M*. This implies that the design should have minimum wall thickness, maximum chamber area, and a cross section that occupies all the available room.

As previously mentioned, a minimal *S*_2_ is desirable. It should be noted that in very specific cases where the reduction of *S*_2_ through material choice has reached the possible minimum, a cross-sectional outline to some degree smaller than the room available may result in a noticeably lower *S*_2_ and therefore improved performance despite the reduction in *pA*. However, these cases are generally unusual, and the performance improvement is typically low as the reduction in ${S_2} / R{S_1}$ is marginal. Hence, the design of a cross section to occupy all available room can be considered a general design principle.

#### Case with *F_n_* + *K* ≠ 0

Considering a case with ${F_n} + K \ne 0$, a similar analysis can be applied. Here, for operation to be viable, ${F_n} + K  <  pA$. Thus, Equation (17) indicates that ${S_2} / R{S_1}$ should be minimized. As in the case with ${F_n} = 0$, Equation (17) indicates that *D* should be maximized while maintaining the values of $\chi$, and therefore, the same stiffness distribution in the region corresponding to *T*_1_ applies. The point of application of *T*_2_, however, is relevant in this case since an equal variation in *D* and $\chi$ can modify *M*. If ${F_n} + K  >  0$, then a high *D* is desirable despite an equal increase in $\chi$. However, a large *A* is also desirable, which can involve a reduction in *D*. Conversely, if ${F_n} + K  <  0$, then a low *D* is desirable, provided that $\chi$ reduces equally. Still, an extensive *A* is also desirable with ${F_n}  <  0$ to maximize the contribution of the integral in Equation (17), which can increase *D* and thereby reduce the performance. In this regard, the most suitable design depends on *F_n_*, *K*, as well as the variation of *D* and $\chi$ with *A* and the geometry. This design problem in the case ${F_n} + K  \ne  0$ in 3D is analogous to that in 2D, but in 3D, an *ad hoc* analysis is required to determine the 3D equivalents of *c*_1_ and *c*_2_ for a given geometry as well as *K* and then generalize the design principles. This involves a numerical study that is beyond the scope of this work; thus, the specific geometry for each configuration under ${F_n} + K \ne 0$ remains as an open question.

#### Final derivation considerations

The derivation with both ${F_n} = 0$ and ${F_n} + K \ne 0$ considered the equilibrium at an arbitrary cross section of the device. As in the planar case, the moment created by the external forces depends on the cross section and therefore can vary along the device. However, the design study is equal despite variations in the external moments and therefore applicable to all cross sections. Thus, the design principles can be applied to all cross sections, defining the most suitable design of the device.

It should be noted that the derivation of the design involves first establishing that the ratio between the stiffness of wall 2 and that of wall 1 needs to be minimized and then determining the geometry. However, in the case that the ratio of stiffnesses could not be minimized, and for ${F_n} = 0$, the design would then need to have an area of the cross section corresponding to the pressurized fluid not occupying all the cross section, which in 2D can be determined from Equation (7). In 3D, this can involve using structures to prevent the cross-sectional deformation, which can justify the introduction of braided chambers in some of the existing designs.^19^

It should also be noted that, as previously discussed, the structure of extending devices is considered to extend only longitudinally, without expanding radially. The introduction of radial expansion would lead to contraction, which is undesirable in extending devices as it reduces the extension. Devices using contraction are discussed in the following section. Extending devices should therefore maintain a constant cross section occupying all available space, which can be achieved by incorporating a set of braces or transversal fibers on the structure of the device.

## Design of Contracting Devices

The deflection in contracting devices is generated by a protruding wall, which forces one side of the device to contract, causing bending of the device. Thus, in contrast to extending devices, the pressure in contracting devices primarily serves to force a wall to protrude, and the moment for bending and supporting external forces and moments is mainly created between the tension in the protruding wall and the compression of another wall. The performance of the device depends on the design geometry and stiffness, which requires a detailed examination.

The design of contracting devices is studied in this section using the same framework as in extending devices. First, the equilibrium of the device is formulated in the Equilibrium section. Energy considerations are then presented in the Energy Considerations section, justifying a set of design principles in terms of the stiffnesses of the device's structure. In the Deflection Condition section, a condition to impose a constant deflection is determined. The equilibrium, energy considerations, and deflection condition are combined in the Design Derivation section to study the design and derive design principles. The design principles to attain a desired deflection with minimum pressure are presented in the Initial Deflection section, leading to the complete design principles for contracting devices, summarized in the Complete Design section. The generalization of the analysis to a 3D scenario is finally described in the Generalization to 3D section.

### Equilibrium

#### Equilibrium formulation

**FIG. 5.**
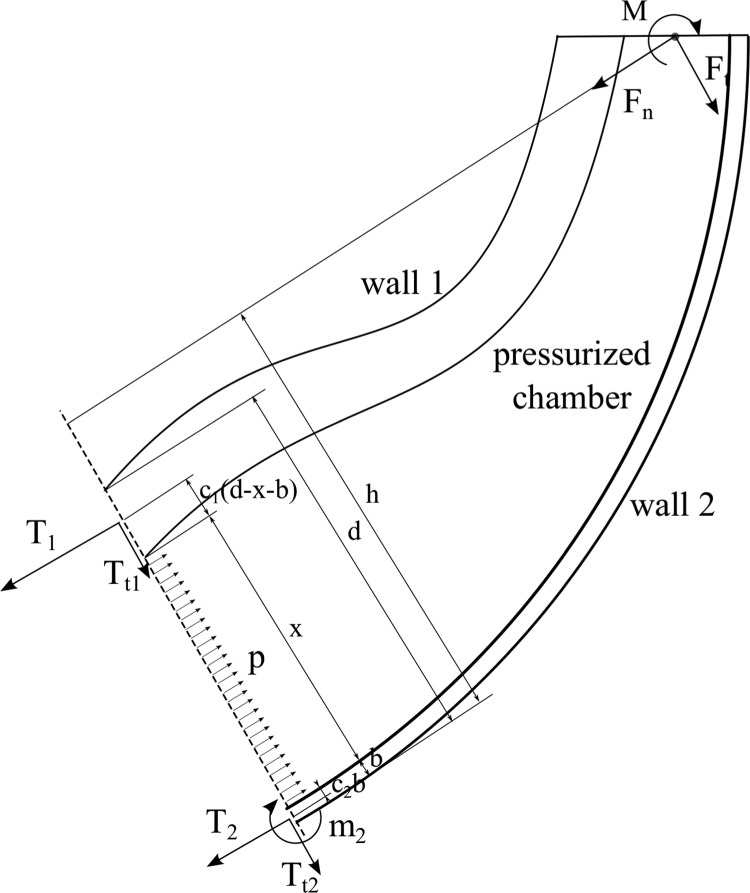
Equilibrium diagram of a 2D contracting device isolated at an arbitrary cross section, exposing reaction forces as well as pressure.

The equilibrium of a general contracting device isolated at an arbitrary cross section can be considered, as illustrated in Figure 5. Imposing equilibrium of forces in the direction orthogonal to the cross section and equilibrium of moments with respect to the point where *T*_2_ is applied, two equations are obtained

\begin{align*}
& { { T_1 } + { T_2 } = px - { F_n } } \\ & { { T_1 } \left[ { {
c_1 } d + x \left( { 1 - { c_1 } } \right) + b \left( { { c_2 } -
{ c_1 } } \right) }
\right]\quad\quad\quad\quad\quad\quad\quad\quad,}
\\ & \quad- \frac { { p { x^2 } } } { 2 } - px { c_2 } b - m_2 + {
F_n } \left( { h - b \left( { 1 - { c_2 } } \right) } \right) = M
\tag { 18 }
\end{align*}

where *b*, *x*, *d*, *c*_1_, *c*_2_, *T*_1_, *T*_2_, ${T_{t1}}$, ${T_{t2}}$, *F_n_*, *M*, and *p* are equivalent to those of extending devices. Equation (18) are analogous to those in extending devices, including the comments on the aggregation of external forces and moments into *F_n_*, *F_t_*, and *M*, as well as the inequalities relating *x*, *b*, and *d* based on geometric constraints.

In contracting devices, wall 1 must protrude and generate a contraction by pulling between its ends, whereas wall 2 must approximately maintain the initial length and bend. Wall 2 therefore serves as a backbone, which may undergo compression stresses. In particular, when ${T_1}  >  px - {F_n}$, wall 2 must be in compression, which typically occurs at low deflections. Hence, the structure of wall 2 typically needs to be capable of supporting compressive stress, and the stress distribution in wall 2 may combine tensioning and compressive stresses. The aggregation of these stresses is decoupled here into a moment associated to bending of the wall, defined as *m*_2_, which can be generally considered to be negative and to reduce further with wall thickness, and the tensioning force *T*_2_. This aggregation of the distributed stresses into *m*_2_ and a normal force *T*_2_ is generally admissible since, as will be seen in the subsequent presentation, wall 2 can generally be considered to act as a rod. The equivalent point of application of *T*_2_ can thus be considered to lay within the wall thickness, $0  <  {c_2}  <  1$, and typically near the center, ${c_2} = 1 / 2$.

#### Equilibrium discussion

As in extending devices, the equilibrium can be considered on any cross section of the device, and therefore, the analysis derived from this equilibrium can be used to study the design of the entire device. Similarly, the equilibrium in the lateral direction also indicates that the structure of the device must support any lateral reactions in a passive manner. However, the effect of shear stresses on deflection is generally negligible and therefore not considered further.

The equilibrium equations (Equation 18) indicate that to maximize the moment that can be supported, *T*_1_ should be maximized and *T*_2_ should be minimized, working in compression. Equation (18) also highlight that the pressure in contracting devices serves two separate purposes. First, and most importantly, it presses on wall 1 to create a protrusion, indirectly contributing to the equilibrium of moments through *T*_1_. Second, it acts on the cross section, directly contributing to the equilibrium of moments as in extending devices. In this regard, contracting devices with equal diameter but different *x* can present different performances and the contribution *px* can be exploited. The direct contribution of *px*, however, also implies a higher tension at the walls, tending to reduce the protrusion, or equivalently limiting *M*, which couples both purposes of pressure.

The design in terms of geometry, including *x* and *b*, and stiffness, predominantly in terms of the protruding wall, must therefore be determined to maximize the *M* that can be supported. Considering Equation (18), configurations that attain high values of *M* in equilibrium with low or even zero *p* can be found. However, each of these equilibrium configurations may correspond to a different deflection. A condition imposing a desired deflection is therefore required to study the effect of design on performance, as in extending devices.

An important difference with respect to extending devices, however, is that in contracting devices the protruding wall is not perpendicular to the cross section along most of the device. The geometry of the protrusion therefore affects the device's performance, and must be first considered, as described in the following section.

### Energy considerations

#### General energetic analysis

The similarities between PAMs and contracting devices imply that some of the existing energetic approaches used in PAMs^12^ can be adapted for the study of contracting devices and thereby extract insight into the behavior of contracting devices. In particular, energetic considerations can be used to elucidate the effect of some aspects of the design, such as structural stiffness, on the performance. Thus, specific aspects of the design, such as stiffness of the protruding wall, can be determined, defining specific protrusion geometries.

Energy conservation must be satisfied in a system corresponding to a general device with a given deflection and supporting general forces and moments. FollowingRef.^12^, virtual works can be considered for the structural deformation caused by a virtual element of fluid *dV* entering the device, with associated virtual increment of displacement *dl* at the point of application and in the direction of the resulting external force *F*, and associated virtual increment of rotation $\theta$ where *M* is applied. Considering an incompressible fluid, this yields

\begin{align*}
pdV = Fdl + Md \theta + d{W_s} , \tag{19}
\end{align*}

where $d{W_s}$ is the work required to deform the structure, which is $d{W_s}  >  0$. Equation (19) elucidates that any $d{W_s}$ reduces the forces and moments that can be supported and therefore should be minimized.

To minimize $d{W_s}$ while maintaining operational capability, the longitudinal stiffness of the protruding wall should tend to infinity. The bending stiffness of the protruding wall, defined *s_b_*, should either be ${s_b} = 0$ over the entire protruding wall or a combination of ${s_b} = 0$ and ${s_b} = \infty$ in different parts of the protruding wall. It should be noted that *s_b_* must be ${s_b} = 0$ at least at some parts of the protruding wall to enable operation. Finally, the bending stiffness of wall 2 should be minimal to minimize $d{W_s}$ or equivalently to reduce the effect of *m*_2_ in Equation (18), but the wall should be capable of supporting compression stresses with minimal contraction.

The energy dedicated to deform the structure can be considered to be practically zero both in designs with only ${s_b} = 0$ in wall 1 and in designs with a combination of ${s_b} = 0$ and ${s_b} = \infty$ in wall 1, which renders both configurations equivalent in this regard. However, Equation (19) also indicates that the forces and moments that can be supported with a given pressure are maximized when the *dV* that corresponds to a pair of *dl*, $d \theta$ is maximized. Thus, the geometry of the protrusion is relevant as it can increase *dV* for a given deflection. The protrusion geometry of designs with only ${s_b} = 0$ in wall 1 is completely determined by the structural behavior. Instead, the protrusion geometry in designs combining ${s_b} = 0$ and ${s_b} = \infty$ depends on the distribution of ${s_b} = 0$ and ${s_b} = \infty$ and can therefore be selected. Considering that a maximum *dV* associated to a *dl*, $d \theta$ at the deflection of operation is desirable, the specific distribution of ${s_b} = 0$ and ${s_b} = \infty$ should be selected to maximize the *dV* associated to an increment of contraction of the protruding wall at the desired deflection, using all available room. This is generally determined geometrically considering that the wall geometry is composed of parts with predetermined geometry corresponding to ${s_b} = \infty$, and parts with a specific geometry corresponding to ${s_b} = 0$, which is a circumference arc as shown in the section Deflection Condition. The constraints from the environment and the desired deflection, however, depend on each scenario, and therefore, the distribution of ${s_b} = 0$ and ${s_b} = \infty$ is specific for each application.

#### Braces and braids

A set of braces can be used as an alternative design option to reduce the protrusion and adapt it to the environmental constraints. The braces need not involve any additional $d{W_s}$ provided that wall 1 is only comprised of parts with ${s_b} = 0$ and ${s_b} = \infty$ and infinite longitudinal stiffness, although the braces should enable wall 1 to protrude to reach the desired deflection. The effect of these braces is thus analogous to that of a wall combining ${s_b} = 0$ and ${s_b} = \infty$, and therefore, they represent an equivalent alternative to select the desired protrusion geometry.

Another design option for wall 1 in 3D scenarios is to include a braided structure such as those used in PAMs,^13^ which may also minimize $d{W_s}$. In particular, a braid that couples longitudinal and transversal tension (and therefore stiffness) through a certain ratio determined by the braid angle may also offer a performance equivalent to that of designs with infinite longitudinal stiffness provided that it requires minimal work to deform it. The braid then simply acts as a mechanism to transform a transversal deformation into a longitudinal deformation. This provides the capability of increasing contraction for a given protrusion, but it requires an in-plane deformation in two directions and thus a 3D structure. Considering that an in-plane extension in the transversal direction is generally not desirable nor practical in soft robotic manipulators with contracting operation and that braids generally involve a certain degree of $d{W_s}$, the use of braids in wall 1 is considered disadvantageous over infinite longitudinal stiffness and therefore not the main focus of this study.

#### Final energy discussion

The design in terms of stiffness can therefore be determined using energetic considerations, as described in previous paragraphs. The energetic considerations, however, do not directly imply a specific design in terms of *x* or *b* since the relation between these and the maximization of *dV*, for a *dl*, $d \theta$ is difficult to determine *a priori*. The equilibrium approach introduced in the section Equilibrium can be used to determine the rest of design and also to develop the study of contracting devices under the same framework as extending devices, but first a deflection condition is required.

### Deflection condition

An incompressible wall 2 is desirable in contracting devices, as argued in previous and following sections. Then, a deflection condition imposing the distance between the ends of wall 1 to remain constant suffices to ensure that deflection is maintained.

#### General deflection condition

The distance between the ends of wall 1 depends on the protrusion geometry and any extension of wall 1. As argued in the section Energy Considerations, a maximal longitudinal stiffness is desirable for wall 1, and therefore, wall 1 can be considered to be inextensible. In this case, the distance between the ends of wall 1 only depends on the protrusion geometry, which is generally a function of *p*, *T*_1_, and *s_b_*. For a given *s_b_*, the distance between the ends of the protruding wall can thus be expressed as $\zeta$, which is a function of *p* and *T*_1_.

The deflection is then determined by $\zeta$. The specific $\zeta \left( {{T_1} , p} \right)$ can be difficult to determine in general as it involves solving a nonlinear structural problem with general boundary conditions. However, considering that $\zeta$, and therefore deflection, depend on the protrusion geometry, insight into the structural behavior of the protrusion can be used to obtain a condition to impose a desired deflection.

In a general protruding wall, an increase in *p* for constant *T*_1_ given *s_b_* leads to a greater protrusion and more contraction, so $d \zeta / dp < 0$. Conversely, an increase in *T*_1_ for constant *p* and *s_b_* tends to reduce the protrusion, hence $d \zeta / d{T_1}  >  0$. Thus, a relation between *T*_1_, *p*, and $\zeta$ generally exists as well for a given *s_b_*, which can be defined as $f \left( {p , \zeta } \right)$. Although $f \left( {p , \zeta } \right)$ is also difficult to determine in general, the function $f \left( {p , \zeta } \right)$ can be either bounded or determined in specific designs of interest, which can suffice to obtain a deflection condition that enables a subsequent design study.

**FIG. 6.**
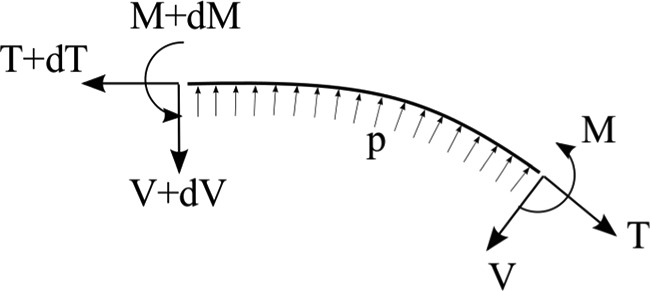
Equilibrium of a differential wall element, where *m*, *V*, and *T* denote the resulting moment, vertical force, and tensioning force at the wall cross section, respectively, and *p* is the pressure that the wall is withstanding.

In particular, in designs with ${s_b} = 0$, the equilibrium of a differential element of wall can be considered, as shown in Figure 6, yielding

\begin{align*}
\begin{matrix} {d{m_1} / dh = {V_1}} \hfill \\ {d{V_1} / dh = p - {T_1}d \psi / dh} \hfill \\ {d{T_1} / dh = {V_1}d \psi / dh} \hfill \\\end{matrix}  , \tag{20}
\end{align*}

where *m*_1_ is the resulting moment at the cross section of the wall, *V*_1_ is the resulting vertical force at the cross section of the wall, and $\psi$ is an angle corresponding to the orientation of the cross section. For ${s_b} = 0$, ${m_1} = 0$. Thus, ${V_1} = 0$, and therefore, *T*_1_ is constant over the wall region, where ${s_b} = 0$. Finally, the relation between *T*_1_, *p*, and the curvature radius of the wall, which can be defined as $R = 1 / d \psi / dh$, is

\begin{align*}
{T_1} = pR. \tag{21}
\end{align*}

The curvature of a wall or part of it over the region where ${s_b} = 0$ is therefore constant. In this regard, the protrusion geometry in designs with purely ${s_b} = 0$ is a circumference arc, whereas the protrusion geometry in designs combining ${s_b} = 0$ and ${s_b} = \infty$ is a combination of circumference arcs and the preselected geometry for the parts with ${s_b} = \infty$. A bijective relation then exists between the geometry of wall 1 and the distance between its ends $\zeta$ in a given design, which is determined geometrically. In particular, in the case of a wall 1 with only ${s_b} = 0$, a certain $\zeta$ implies a specific *R*. In the case of a wall 1 combining ${s_b} = 0$ and ${s_b} = \infty$, a given $\zeta$ also implies a certain *R* that is common in all regions where ${s_b} = 0$ and is generally lower than the *R* in designs with only ${s_b} = 0$ for an equal $\zeta$.

The wall curvature is directly related to *T*_1_ and *p* according to Equation (21). Hence, a pair of *T*_1_ and *p* imply a protrusion geometry, which in turn entails a certain $\zeta$ and can therefore be used as a condition to impose a desired deflection. Equivalently, using the relation between *R* and $\zeta$ described in the previous paragraph for each particular design, Equation (21) can be transformed into the condition

\begin{align*}
f \left( {p , \zeta } \right) = pR \left( \zeta \right) , \tag{22}
\end{align*}

where $R \left( \zeta \right)$ is determined geometrically. Thus, for a given design in terms of distribution of ${s_b} = 0$ and ${s_b} = inf$, the tension in wall 1 to maintain a desired deflection is proportional to *p* and determined by Equation (22) with the *R* corresponding to the regions, where ${s_b} = 0$. The condition applies to the entire wall. This includes regions where ${s_b} = \infty$ since the moment at the ends of these regions is zero, and therefore, the tension and its line of application within these regions are constant and equal to the tension at the ends.

The result that the wall geometry is specific for a certain distance between the ends of a protruding wall in wall designs with ${s_b} = 0$, and infinite longitudinal stiffness is coherent with the energetic considerations. Indeed, if deformation energy cannot be stored in the structure, an increase in *p* and *T*_1_ that maintains the distance between the ends of the wall and thus involves no motion of the device cannot result in any change in the geometry to satisfy energy conservation.

#### Particular designs with braces or braids

In designs including a set of braces, the deflection condition is similar. However, the specific design of the braces can lead to different values of tension in each segment of wall between two braces, particularly if the braces are not perpendicular to the cross section, resulting in different curvatures at each segment of the protrusion. The effect of the braces on the resulting wall tension must therefore be considered to then use condition (Equation 22) with the corresponding *R*. This effect can be determined by considering equilibrium at the point of attachment of the braces. However, it is not developed in this work since braces simply represent an alternative to modify the protrusion geometry equivalent to designs combining ${s_b} = 0$ and ${s_b} = \infty$ but do not provide specific performance advantages, as discussed in the section Energy Considerations. It should be noted, however, that braces typically involve a reduction in *R*, which entails lower *T*_1_ and therefore lower force for a given *p* and deflection, but also lower protrusion magnitude.

A deflection condition similar to Equation (22) can also be obtained in designs with braids, provided that ${s_b} = 0$ is a valid assumption for the braid. However, condition (Equation 22) is derived considering a planar case, and a direct generalization to 3D only applies to protrusions with bending in a plane. Braids, however, couple transversal and longitudinal deformations and therefore are intrinsically 3D. Considering that the use of braids is generally disadvantageous as discussed in the section Energy Considerations, and that the generalization of this study to 3D is discussed in the Generalization to 3D section, the deflection condition for designs with braids is not considered further in this section.

### Design derivation

The equilibrium equations (Equation 18) and the deflection condition (Equation 22), together with the energetic considerations, can be combined to study the design and to derive design principles to maximize the forces and moments that can be supported.

#### Detailed analysis and derivation

First, the energetic considerations described in the Energy Considerations section can be used to determine the wall stiffnesses of the design. In particular, the design should generally have an inextensible protruding wall with either ${s_b} = 0$ or a combination of ${s_b} = 0$ and ${s_b} = \infty$ to maximize the *dV* associated to a *dl*, $d \theta$ at the desired operation deflection, using all available room. The specific combination of ${s_b} = 0$ and ${s_b} = \infty$ depends on each specific application, but typically larger regions of ${s_b} = \infty$ provide higher *dV* and thus higher performance at low deflections, whereas larger wall regions with ${s_b} = 0$ enable reaching and providing some support of external forces and moments at larger deflections. With these stiffnesses, the tension of the protruding wall (Equation 21) can be combined with the equilibrium of forces (Equation 18) to show that wall 2 must be designed to be capable of supporting compressive stress to allow operation at low deflections where *R* tends to infinity.

In these designs of interest, the equilibrium equations (Equation 18) can then be considered, and a desired deflection can be imposed by substituting Equation (22), yielding

\begin{align*}
& pR(\zeta) { \rm{cos}} \alpha [ {{c_1}d + x ( {1 - {c_1}} ) + b (
{{c_2} - {c_1}} ) } ] \\ & - p{{{x^2}} \over 2} - px{c_2}b - {m_2}
+ {F_n} ( {h - b ( {1 - {c_2}} ) } ) = M ,
 \tag{23}
\end{align*}

where $\alpha$ is the angle between the direction of the resulting tensioning force in wall 1 and the direction normal to the plane of the cross section. Equation (23) elucidates the fact that the design to maximize *M* depends on $\alpha$ and therefore on the protrusion geometry. This is determined by the aforementioned stiffnesses, which maximize performance as discussed in the Energy Considerations section and thus $\alpha$ is a specified value at each cross section.

Equation (23) provides the relation between the *M* that can be supported at a desired deflection and the design. Equation (23) is analogous to Equation (6) in extending devices and can therefore be used to derive the additional design principles to meet the objective of maximizing *M*. Equation (23) is valid in general and thus enables the determination of the design in a general scenario.

#### Case with *F_n_* = 0

A case with ${F_n} = 0$ can be studied first, as it represents a common scenario of interest in practice, and is illustrative of the design principles. The effect of *x*, *b*, and *d* on Equation (23) is relatively decoupled in the majority of terms. However, their contributions to *M* depend on the values of *c*_1_ and *c*_2_, especially in terms of $sgn \left( {c2 - c1} \right)$, and therefore, these two parameters must be first considered.

Specific values of *c*_1_ and *c*_2_ can be difficult to select with the design, but general tendencies for the desired values of the parameters can be considered, which can suffice for the design study. The value of *c*_1_ affects *M* through three terms: a positive and two negative ones. However, considering that the variables *x*, *b*, and *d* are related through $x + b  <  d$, it can be seen that the total contribution of *c*_1_ to *M* is always positive, and therefore, *c*_1_ should be maximized to the extent it is possible with the design. The contribution of *c*_2_ to *M* is more complex to analyze, and therefore, its desired tendency is difficult to determine. However, considering that *c*_1_ and *c*_2_ are equivalent design parameters, their maximum values can be considered similar. Thus, in a design where *c*_1_ is maximized, it can be assumed that ${c_2}  <  = {c_1}$.

The $sgn \left( {{c_2} - {c_1}} \right)$ can then be considered to be negative. This implies that *b* tends to reduce *M* in Equation (23). In addition, *b* also tends to reduce *m*_2_, leading to more negative values, which reduces further *M*. Thus, *b* should generally be minimized, which can be expressed as $b = 0$.

The contribution of *x* to *M* in Equation (23) is then only through two terms, with a quadratic relation. Thus, the value of *x* to maximize *M* can be directly determined as $x = \left( {1 - {c_1}} \right) R \left( \zeta \right)$. Considering the constraint $x  <  d$, the value of *x* should tend to *d* at low deflections, where $R{ \rm{ \;}}  \to { \rm{ \;}}  \infty$, even for a *c*_1_ that is maximized, which can be expressed as $x = d$. At larger deflections, the required value of *x* may be lower than *d*. A design where *x* reduces with deflection can be considered to be inviable in practice unless pressures below atmospheric pressure are used, which is typically impractical as it limits the maximum pressure difference. Thus, at larger deflections, the design in terms of *x* should be selected for a specific operation according to $x = R \left( \zeta \right) \left( {1 - {c_1}} \right)$.

Finally, the value of *d* is determined by the environment in each application. Equation (23) shows that a high *d* is desirable, and therefore, it should be the selected so that the device reaches the constraints from the environment at the maximum protrusion. This agrees with the aforementioned design of wall 1 to use all available room, although the specific wall geometry, determined by the regions with ${s_b} = 0$ and ${s_b} = \infty$, should be selected to maximize the *dV* associated with *dl*, $d \theta$, as previously described.

#### Case with *F_n_* ≠ 0

The design in the general case ${F_n} \ne 0$ can be studied in a similar manner. In contracting devices, the deflection condition (Equation 22) only imposes a constraint on *T*_1_ but does not involve *T*_2_. As a consequence, the contribution of *F_n_* to *M* in Equation (23) is through a constant term ${F_n}h$ and a term depending on the design ${F_n}b \left( {1 - {c_2}} \right)$. As in extending devices, the contribution of the term ${F_n}b \left( {1 - {c_2}} \right)$ to *M* can be disregarded since it is equivalent to offsetting the device with respect to the external forces. The contribution ${F_n}h$ is fixed and equivalent to an additional external moment to be supported.

The design derivation in the case ${F_n} \ne 0$ is therefore analogous to that in the case ${F_n} = 0$, and the principles for contracting devices both with and without external forces and moments are equivalent. These, together with the aforementioned principles corresponding to the stiffnesses of the walls, constitute the design principles to maximize the *M* that can be supported at a given deflection with contracting devices.

#### Final derivation considerations

It should be noted that in designs combining ${s_b} = 0$ and ${s_b} = \infty$, the derivation also applies to the regions, where ${s_b} = \infty$. However, in these regions, both the line of application of *T*_1_ and $R \left( \zeta \right)$ correspond to those at the boundaries with the adjacent regions, at the side where ${s_b} = 0$. Thus, the line of application of *T*_1_ needs not necessarily be within the wall in designs with curved rigid wall regions. The design principles, however, indicate that *d* should be maximized within the room available and *x* should generally occupy the entire cross section. Hence, the rigid parts in wall 1 should be straight, and the same principles derived in previous paragraphs apply.

Interestingly, the geometric principles indicate that the thickness of wall 1 and wall 2 should be minimized in the majority of cases. This is coherent with the principles in terms of stiffness, indicating that the bending stiffness of wall 2 should be minimal while supporting compression stress and the bending stiffness of wall 1 should be minimal in the desired regions. Thus, the resulting designs can be produced in practice.

#### Derivation discussion

This derivation confirms that wall 2 in standard contracting devices must undergo compressive stress when $R \left( \zeta \right)  >  = x - {F_n} / p$, which typically occurs at low deflections. High values of *x* can aid in reducing the compressive stress, but, in general, designs without the capability of supporting compressive stress in wall 2 cannot operate at low deflections. This is due to the fact that a protrusion generally involves a *T*_1_.

The need for a wall 2 capable of supporting compressive stress can only be prevented by reducing the *T*_1_ associated with a protrusion and *p*, which requires exceptional solutions. One of such solutions is to include an elastic sheet that acts as a continuous set of elastic braces opposing to the protrusion, thereby reducing *T*_1_ and thus leading to a contracting device without the need for a wall 2 capable of supporting compressive stress. Such a design solution is relevant in the application described in the Summary section. However, in general, such a solution also involves a reduction in the forces and moments that can be supported.

### Initial deflection

The design principles to attain a desired initial deflection with minimum pressure can be determined by following a similar derivation as that to derive the principles to maximize the forces and moments that can be supported.

First, the energetic considerations described in the Energy Considerations section indicate that the structure should store minimum energy. This implies an inextensible protruding wall with either ${s_b} = 0$ or a combination of ${s_b} = 0$ and ${s_b} = \infty$. Wall 2 should then be incompressible and with minimum bending stiffness.

The study of the protrusion in the Deflection Condition section indicates that the protrusion geometry is directly related to the deflection. Thus, the desired initial deflection can be imposed by selecting a protrusion geometry with a desired *R* and using the deflection condition (Equation 22), where the specific $R \left( \zeta \right)$ is determined geometrically.

Equilibrium of moments can also be considered in a device at the desired initial deflection and with no external forces or moments. This is equivalent to the equilibrium in Equation (18), shown in Figure 5, particularized to ${F_n} = 0$, $M = 0$. The imposition of the desired initial deflection (Equation 22) to Equation (18) yields

\begin{align*}
p { R_i } \left( { { c_1 } d + x \left( { 1 - { c_1 } } \right) + b \left( { { c_2 } - { c_1 } } \right) } \right) - \frac { { p { x^2 } } }  { 2 } - px { c_2 } b - { m_2 } = 0. \tag { 24 } 
\end{align*}

This equation can be used to determine the design to attain the desired initial deflection with minimum pressure. First, Equation (24) indicates that to minimize *p*, *m*_2_ should be minimized, which agrees with the energy considerations. Then, factorizing *p*, it can be seen that the design to minimize *p* in Equation (24) is equivalent to the design to maximize *M* in Equation (23). Hence, the design geometry and stiffness should be equal to those derived in the section Design Derivation.

### Complete design

The design principles to attain a desired deflection at minimum pressure are equal to those to maximize the forces and moments that can be supported at a desired deflection, both with and without *F_n_*. Thus, these represent the general design principles for contracting devices and are summarized subsequently.

The protruding wall should have infinite longitudinal stiffness and either ${s_b} = 0$ or a combination of ${s_b} = 0$ and ${s_b} = \infty$ to maximize the *dV* associated with an increment in the contracting wall, using all available space. Wall 2 should be incompressible with minimum bending stiffness. The parameter *c*_1_ should be maximized to the extent possible. The total width *d* should be selected so that wall 1 reaches the constraints from the environment at maximum protrusion. And finally, the design geometry should be $b = 0$ and $x = R \left( \zeta \right) \left( {1 - {c_1}} \right)$, which is typically $x = d$.

### Generalization to 3D

The design derivation presented up to this point can be generalized to 3D. The study in 3D is mostly equivalent: it involves using energy considerations to outline the device's stiffness and then combining it with an equilibrium analysis to derive the design principles. However, some aspects of the generalization require a detailed analysis.

#### Generalization of design derivation to 3D

The energy considerations described in the Energy Considerations section can be applied to 3D, showing that the structure of a 3D device should store minimum energy to maximize the forces and moments that can be supported. Thus, the structure of a 3D contracting device must be composed of two regions: a first region corresponding to a protruding wall, which should be inextensible and with either ${s_b} = 0$ or a combination of ${s_b} = 0$ and ${s_b} = \infty$, and a second region of the device acting as a backbone, which should be incompressible and with minimum bending stiffness, equivalent to wall 2 in the planar case.

The cross section of the device must then be divided, with parts corresponding to these two structural regions. Unlike in extending devices where the role of the cross-sectional stress in the cross section is dictated by the position relative to the center of pressures, in contracting devices, the purpose of the local stress in each element of area over the cross section is not clear *a priori*.

A cross section divided along an arbitrary curve can be considered. This defines the two regions in terms of stiffness, where one region corresponds to the protruding inextensible wall and the other region corresponds to the incompressible wall. The equilibrium of the 3D device isolated in this general cross section divided along an arbitrary curve can then be considered in an analogous manner as described in the Equilibrium section, with *T*_1_ corresponding to the aggregated normal stresses in the region of the protruding wall and *T*_2_ corresponding to the aggregated stresses in the other region. The equilibrium indicates that to maximize the forces and moments that can be supported, the separation between *T*_1_ and *T*_2_ should be maximized. Thus, the curve dividing the cross section must be selected to maximize the distance between *T*_1_ and *T*_2_ in the direction perpendicular to these forces and in the plane of bending. This specifies the purpose of each region of the cross section and defines the stiffnesses of the device.

Equilibrium of the 3D device isolated in an arbitrary cross section with *T*_1_ and *T*_2_ defined by this dividing curve can be used to determine the rest of the design in an equivalent manner as in the planar case. The design involves minimizing the thickness of the region corresponding to *T*_2_, maximizing the area of the cross section corresponding to the pressurized chamber for typical operation deflections, and maximizing the increment of volume in the device for an increment in contraction of the protruding wall at the operation deflection using all available space.

**FIG. 7.**
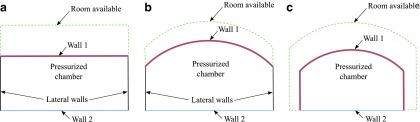
Diagrams of a typical cross-sectional design in a scenario with rectangular constraints **(a)**, typical cross-sectional design in a general scenario with curved constraints **(b)**, and undesirable cross-sectional design in general scenario **(c)**. In all diagrams, wall 1 is depicted in *red*, wall 2 in *blue*, lateral walls in *black*, and the available room in the scenario in *dashed green lines*.

The specific division of the cross section along a curve, or equivalently the allocation of the different parts of the cross section to the different regions, to maximize the distance between *T*_1_ and *T*_2_ depends on each scenario. In typical scenarios where the spatial constraints in a cross section are defined by a rectangle, wall 1 should correspond to one side of the rectangle and wall 2 to the opposite side, as shown in Figure 7a. In more general scenarios with any spatial constraints, wall 1 should correspond to the entire frontal region of the device when observed from the direction in which it bends, as illustrated in the example in Figure 7b, creating a frontal protrusion, while wall 2 should correspond to the opposite side.

These designs oppose to designs with a wall 1 that extends to the lateral regions, such as that shown in Figure 7c. Protrusion in the lateral direction, or in any direction different from a frontal protrusion, is generally undesirable. This can be elucidated using the equilibrium, as it generally involves increasing the region corresponding to *T*_1_ to the laterals, which modifies the line of application of *T*_1_, reducing the distance between *T*_1_ and *T*_2_. The undesirable lateral protrusions can also be explained using energetic considerations. The forces and moments that can be supported depend on the volume increase of the device (Equation 19) for a contraction increment. However, the geometry of the protrusion generally cannot be selected to adapt exactly to the volume available from the spatial constraints, which are commonly prismatic, leaving some volume unused. In designs with lateral protrusions, the unexploited volume is typically larger than in designs with only frontal protrusion, as unused volume appears at both sides or near vertices of the available room, leading to lower performance. Thus, both equilibrium and energetic considerations confirm that the protrusion should generally be only frontal.

Designs in 3D, such as those shown in Figure 7a and b, typically include lateral walls. However, these should not contribute to the protruding wall nor to the opposite wall to maintain the distance between *T*_1_ and *T*_2_ to a maximum. These lateral walls only serve to contain the pressurized fluid and enable protrusion of wall 1 but should not affect the structural behavior of the device. Thus, these walls should generally be designed to minimize any resistance to deformation while containing the fluid without protruding laterally, for example, using a pleated structure with tendons connecting both laterals. In specific cases, however, these lateral walls can be used to reduce the *T*_1_ associated to a deflection and pressure, reducing the compression on wall 2. This is equivalent to the use of an elastic sheet introduced in the Design Derivation section for planar designs and is a relevant solution in the design presented in the following section.

#### Discussion of 3D derivation

The design of contracting devices in 3D presented in this section elucidates that contracting devices are similar to a segment of continuum robot actuated by PAMs and with an elastic backbone, such as Ref.^30^ However, contracting devices integrate different parts and can be designed with the principles elucidated in this work to improve performance. Still, both contracting devices and devices including PAMs present the disadvantage of involving a protruding wall, which typically protrudes outward, requiring additional room to operate.

## Summary

The main design principles derived in the previous sections are summarized in the Extending Devices section for extending devices and then in the Contracting Devices section for contracting devices. The overall procedure to design a soft robotic manipulator using the design principles is then outlined in the Outline of Design Principles Application section. It should be noted that this section is intended as a summary of the main principles, and the reader is referred to the previous sections for details and clarifications on the principles and their derivation.

### Extending devices

The design principles for extending devices in both 2D and 3D can be summarized as follows. The longitudinal stiffness in the region corresponding to wall 1 should be maximized, which can be expressed as maximal *s*_1_ in 2D and equivalently maximal *S*_1_ in 3D. The stiffness distribution should be selected so that the maximum stiffness is concentrated near the edge of the cross section in the direction of bending to displace the line of application of *T*_1_ toward the cross-sectional contour. The stiffness in the region corresponding to wall 2 should be minimized, which can be expressed as minimal *s*_2_ in 2D and minimal *S*_2_ in 3D. This minimal stiffness can be achieved, for example, with a pleated structure. The total cross section of the device should be maximized to occupy all available room. The thickness of the walls should be minimized. Finally, the chamber area should be maximized, in general case where ${S_2} / R{S_1}$ is minimized, to ensure that the region of the cross section corresponding to the pressurized fluid is maximal. It should be noted that these last three principles apply to any 2D case and to the 3D case ${F_n} + K = 0$. However, in the 3D case ${F_n} + K \ne 0$, the specific geometry of the cross section must be determined using numerical methods.

The performance of extending devices is related to their operation. In extending devices, the combination of *T*_1_ and the direct contribution of pressure in the cross section create the moment that supports external moments and equivalent moments generated by external forces. Thus, the performance of extending devices tends to be relatively low at low pressures but remains relatively constant as deflection and pressure increase. As a result, extending devices are relatively well suited to operate at large deflections and corresponding higher pressures.

### Contracting devices

The design principles for contracting devices in both 2D and 3D can be summarized as follows. The longitudinal stiffness of the protruding wall should be maximal. Its bending stiffness should generally be a combination of parts with infinite and minimal bending stiffness, selected to maximize the *dV* corresponding to an increase in wall contraction at the operation deflection, although in specific cases, braids or braces can be used to maximize the *dV* associated with a contraction increase. Wall 2 should be capable of bending with minimum resistance while generally being capable of supporting compression forces. The distance between *T*_1_ and *T*_2_ should be maximized by selecting appropriate regions for walls 1 and 2, as illustrated in Figure 7. This implies that in some cases lateral walls may be included, typically in the form of pleated structures with braces to prevent lateral expansion. However, these lateral walls should only serve to contain the pressure and not affect the structural behavior of the device. The total cross section should be maximized so that the device occupies all available room at the operation deflection, where the protrusion should be maximal. The thickness of the walls should be minimized. Finally, the region of the cross section with pressurized fluid should generally be selected to be maximal at the operation deflection.

The performance of contracting devices is also related to their operation. The support of external moments and equivalent moments generated by external forces is primarily achieved between wall 1, which is in tension thanks to the pressure forcing wall 1 to protrude and wall 2 in compression. The direct contribution of pressure to the moment at the cross section is then secondary. As a result, their performance is relatively high at low deflections, where low pressures produce significant *T*_1_, but tends to reduce at higher deflections, where the *T*_1_ created by a given pressure is lower. This behavior is analogous to that of PAMs.^12^

### Outline of design principles application

**FIG. 8.**
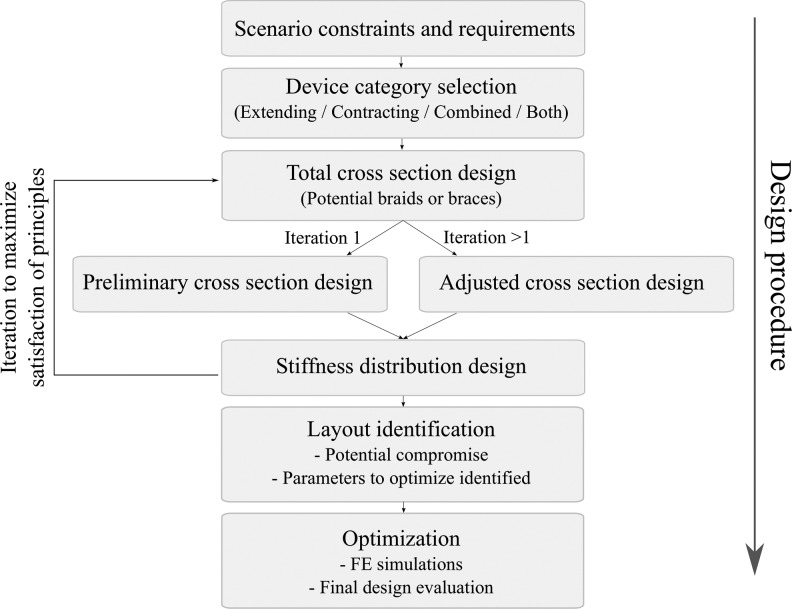
Flowchart outlines the overall procedure to design a soft robotic manipulator in a given scenario. The chart summarizes the main design steps, which are implemented using the design principles derived in this work.

The design principles can be used in the process of determining the most suitable design in each scenario. The design depends on multiple factors in terms of requirements and constraints of the scenario, so each case needs to be considered individually. Nonetheless, an overall design procedure exists, which is generally common. This is schematized in Figure 8 and outlined subsequently.

First, the spatial constraints and the scenario requirements (typically desired deflection) are considered, and the category of device is selected accordingly. If the desired deflection is relatively low and some space is available for a protrusion, a contracting device is selected. Conversely, if the desired deflection is high or the maximum diameter is very constricted, an extending device is selected. If the desired deflection presents a broad range of values of interest, a device combining extending and contracting actuation can be selected. Finally, if the desired deflection is intermediate, both an extending device and a contracting device need to be explored, and the most suitable design needs to be selected by comparing the performance of the final designs of both types of devices.

Once the type of device is chosen, the total cross section is selected to occupy all room available at the desired deflection, as indicated in Figure 8. In some cases, braids or braces may be introduced to adapt to the total cross section to the spatial constraints. A preliminary cross-sectional geometry is then designed, following the design principles and defining a preliminary estimate of the regions corresponding to each wall.

The stiffness distribution is then selected, following the design principles. In most contracting devices, this can affect the design of the total cross section to use all available room and any braids or braces associated with it, and thus, they need to be designed in conjunction. Once the stiffness distribution and total cross section are established, the cross-sectional geometry is adjusted according to the design principles. Iteration can then be conducted to satisfy all design principles to the best possible extent, as shown in Figure 8.

The design procedure up to this point provides the most suitable design layout. In some cases, the design principles can show that a compromise is necessary, as not all principles can be concurrently satisfied. In addition, the value of specific design parameters may need to be optimized, which is also generally identified by the design principles. FE simulations can be used to optimize the parameters, and resolve the compromises, yielding the final design. The FE simulations can also be used to compare final performance of designs in the case that both an extending device and a contracting device are explored and thus select the best. An example of design application is presented in the following section, which showcases this design procedure in a problem that illustrates the different steps, described subsequently.

## Application to Manipulator Design

The design principles distilled in the previous sections (Design of Extending Devices; Design of Contracting Devices; and summarized in section Summary), are applied in this section to derive the design of a soft robotic manipulator in a prototypical scenario.

### Scenario definition

A MIS scenario requiring a soft robotic manipulator is selected as the prototypical scenario in this work. Soft robotic manipulators are well suited to MIS, offering compliance, modularity, compatibility with magnetic resonance imaging, and miniaturization possibilities that are particularly desirable in keyhole surgery. The recent interest in the subject^3^ illustrates the relevance of these devices in medical applications.

The specific requirements for the soft robotic manipulator in the selected scenario are for it to be able to bend laterally in any direction, providing two degrees of freedom, and to maximize the lateral force that can be supported at deflections near 20°. This deflection is measured as the angle between the centers of the manipulator's ends in undeformed and deformed configurations and is selected arbitrarily to illustrate the determination of the design in a representative case. The outer diameter of the device is constrained to 6 mm, and the operation pressure is limited to 6 psi. These are typical values in MIS where a small diameter is required for entry into the body, and the maximum pressure is limited due to the relatively weak sealing at miniature size and to prevent damage in case of bursting. These values are also similar to the pressures and deflections considered in the literature for devices with similar characteristics.^31,32^

The minimum wall thickness is considered to be limited by manufacturing constraints and associated resilience to puncture, leakage, and withstanding the maximum pressure. The manufacturing of soft robots commonly involves casting the hyperelastic structure of the device, adding fibers, sheets or other inextensible elements, and finally affixing all the elements typically with additional layers of hyperelastic material. Considering the typical tolerances associated with these processes, a minimum wall thickness of 400 μm is selected for the prototypical scenario. The suitability of this thickness to withstand the maximum pressure with a safety margin to cope with manufacturing tolerances while providing a certain degree of resilience is confirmed in the simulations in the Application to Manipulator Design section.

### Design derivation

#### Primary design derivation

The principles of operation of extending and contracting devices are different, which makes the devices suitable for operation at different deflections. Contracting devices predominantly support external forces and moments thanks to the pressure forcing the protruding wall to be in tension and thus the opposite wall in compression, and the direct contribution of pressure to support external moments is secondary. As a consequence, they generally offer higher performance at lower deflections where even low pressures create significant tension in the protruding wall. However, as deflection increases, the relation between *T*_1_ and *p* reduces, and *T*_2_ becomes a tension force, leading to lower performance. Conversely, extending devices support external forces and moments thanks to the direct contribution of pressure to generate a moment when considering equilibrium of a device isolated in a general cross section, in combination with *T*_1_. Thus, they typically offer lower performance at low deflections and low pressure, but their performance remains relatively constant as deflection and pressure increase, offering higher performance at higher deflections. A design combining extending and contracting operation would therefore be advantageous in this application that requires operation at various deflections.

The design principles for extending and contracting devices share many similarities. The wall thickness should generally be minimized, and the area of the cross section corresponding to the pressurized fluid should be maximized; the devices should use all available room; the region corresponding to wall 1 should present a maximal longitudinal stiffness, and this should be concentrated near the edge to maximize the distance between the line of application of *T*_1_ and *T*_2_. In addition, these principles are generally independent of the desired deflection and pressure. Thus, a design combining both types of operation can be conceived for this scenario.

The main design difference is that, in extending devices, a wall 2 with minimum longitudinal stiffness is desirable, as elucidated in the Design of Extending Devices section, which can be attained with a pleated structure. Instead, in contracting devices, wall 2 must typically support compressive stresses, as shown in the Design of Contracting Devices section, and therefore, a pleated structure is not viable. Thus, a certain degree of compromise is necessary.

In this prototypical scenario, any protrusion over 6 mm diameter is undesirable. Thus, the outer structure should be cylindrical with 6 mm diameter. To provide bending in any direction, the design must be 3D and should then include at least three chambers in the cross section along the device. Since chambers involve partition walls that increase bending stiffness, the number of chambers should be minimized, leading to three chambers being selected. The design principles indicate that the cross-sectional deformation is desirable from an extending device perspective to maximize the area of the cross section corresponding to the pressurized chambers and displace the line of application of *T*_1_ toward the outer contour, with maximum concentration of stiffness at the region corresponding to *T*_1_. Such cross-sectional deformation leads to a protruding central rod. This can be exploited as the protruding wall in contracting devices. Thus, the central rod should have infinite longitudinal stiffness, which is desirable for it to act as the protruding wall of extending devices, and as wall 1 of extending devices. This results in a device combining extending and contracting operation, with a design that is desirable for both types of operation as it maximizes area of the cross section corresponding to the pressurized fluid and presents a desirable stiffness at the equivalent of wall 1.

Since the design includes contracting operation, a structure capable of supporting compressive stress is necessary to act as wall 2. The device must be capable of bending in any direction; hence, the line of application of the equivalent of *T*_2_ must be near the center of the device. The most suitable solution is then the incorporation of an outer cylindrical structure made of superelastic material such as nitinol, with notches in alternating perpendicular directions to enable bending with minimum resistance while supporting compression forces.

**FIG. 9.**
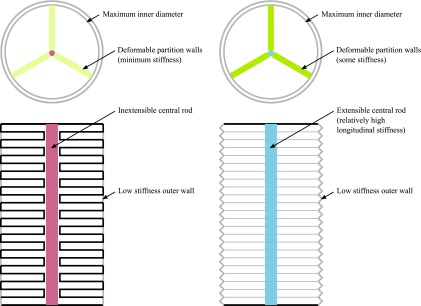
Conceptual illustrations of the most suitable design (*left*) and alternative design (*right*). The design on the *left* includes three partition walls with minimal stiffness to facilitate the cross-sectional deformation, an inextensible central rod, a minimal outer wall thickness of 400 μm made of low-stiffness material, and a notched outer structure to support compression force while minimizing resistance to bending. The design on the *right* also has an outer wall with minimal thickness and minimal resistance to bending, but it does not include an outer structure, and instead, it has outer fibers to prevent the radial expansion. In addition, its three partition walls are also deformable but present some stiffness, which combines with a central rod that can extend to some degree to prevent compression of the outer wall.

The most suitable design of the soft robotic manipulator is therefore a cylinder with a constant cross section that consists of three equal chambers that can deform and present a maximum area, and an outer metallic structure, as conceptually illustrated in Figure 9 (left). The ratio ${S_1} / {S_2}$ should be maximized according to the design principles, which implies minimal stiffness at the outer wall and maximal longitudinal stiffness at the central rod. This can be obtained by designing an outer wall made of minimal stiffness material and with minimum thickness, which in this scenario corresponds to 400 μm as described in the previous section, and including an inextensible thread at the central rod. The stiffness of the partition walls should be minimal to facilitate the cross-sectional deformation, and wall thickness should be minimal to maximize the area of the chambers in the cross section. Since the maximum cross-sectional deformation is limited by the outer wall, the partition walls are always below the maximum strain of typical hyperelastic materials, and thus, the minimum wall thickness in this scenario, 400 μm, can be selected. It should be noted that the outer structure serves to prevent radial expansion of the outer wall. The maximum protrusion of the central rod is also limited by the outer diameter, and therefore, the device respects the diameter constraints while offering contracting operation.

#### Discussion of primary design found

The design layout of this primary design obtained resembles that of the FMA, but the principles of operation and the specific geometry and stiffnesses are different. This layout combines extending and contracting operation, in contrast to the FMA that only involves extending operation. In addition, the wall thickness in this layout is lower than that in the FMA to maximize the chamber area in the cross section, the central rod is inextensible to maximize force, and the design includes an outer structure to support compression forces. Finally, the partition walls in the proposed layout contrast with those in the FMA, as they are designed to facilitate the cross-sectional deformation, which maximizes the area corresponding to the pressurized fluid in the cross section and leads to contracting operation.

The manufacturing of the proposed outer structure capable of supporting compression forces is challenging, particularly at the miniature size of this prototypical scenario. In addition, it can introduce bending resistance, limiting performance. Furthermore, the structure can limit extending-type operation at relatively high pressures.

#### Alternative design

An alternative design without the outer structure can be conceived, which is easier to manufacture and more illustrative of the research presented in this article, enabling the verification of some of the design principles. The need for a structure to support compressive forces stems from the significant tension at the central rod associated with a protrusion and mainly occurs at low deflections. As mentioned in the Design Derivation section and in the Generalization to 3D section, this tension can be reduced by introducing some resistance to the protrusion. In this 3D design, the resistance can be introduced by using partition walls with some stiffness. Thus, by combining partition wall stiffnesses (PWS) together with some extension of the central rod, a design without compression force on the outer structure can be achieved. It should be noted that, in such design, the central rod still serves to increase performance by introducing contracting actuation and by maximizing ${S_1} / {S_2}$; hence, a high central rod stiffness in the longitudinal direction is desirable. The main purpose of the partition walls is to compensate any excessive effect of the protrusion for a given pressure and deflection, and therefore, the PWS depends on the longitudinal central rod stiffness (LCRS), with higher LCRS requiring higher PWS.

The alternative design is therefore similar to the previous design, as conceptually illustrated in Figure 9 (right), but with different values of PWS and LCRS. This leads to a design without compression at the outer wall, which eliminates the need for complex structures while enabling operation at low deflection. Consequently, it represents the design selected for this prototypical scenario.

The outer wall can then be made of soft material, and according to the design, principles should have a minimal wall thickness to maximize the area of the cross section corresponding to the pressurized fluid and a minimal bending stiffness to maximize ${S_1} / {S_2}$. A pleated structure with circumferential fibers could be used to minimize bending stiffness, but manufacturing at millimetric scale can be challenging. Instead, a cylindrical outer wall made of soft material with circumferential fibers to prevent radial expansion while allowing the longitudinal deformation is practically equivalent and easier to manufacture, hence is the solution selected. The material of the outer wall should be hyperelastic, with low stiffness, and capable of withstanding pressure when combined with fibers. To consider a realistic material that is readily available, Dragon Skin 10 (Smooth-On) is selected for this prototypical scenario. This is a common material in soft robotics and it has been previously characterized in the literature.^33^ The wall thickness should be the minimum possible, which corresponds to 400 μm in this scenario, as described in the previous section. This wall thickness can withstand ${p_{max}}$ with only minor bulging of the rubber between the fibers, which corresponds to a maximum strain in the rubber below the failure limit of the material, as confirmed in the simulations in the section FE Simulations. The cross-sectional area corresponding to the pressurized fluid should also be maximized according to the design principles, which implies a minimum partition wall thickness of 400 μm. This principle also implies that the cross-sectional deformation is also desirable, which however can be limited by PWS. Thus, the contributions of PWS and LCRS need to be matched to achieve the desired performance.

#### Compromise in optimal design parameters

**FIG. 10.**
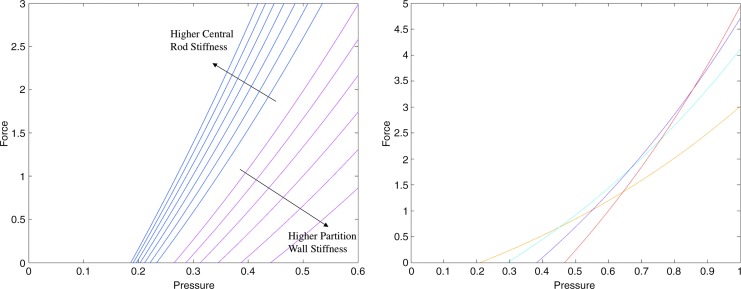
(*Left*) Qualitative graph illustrating the design trends corresponding to the variation of LCRS in a device where the rest of the design remains equal, shown in *blue*, and the variation of PWS in a design where the rest remains equal, shown in *magenta*. (*Right*) Qualitative graph illustrating the performance of designs optimized for different ${p_{max}}$ in terms of their PWS and LCRS, elucidating the fact that the design parameters PWS and LCRS must be optimized for the specific pressure of operation in each scenario. LCRS, longitudinal central rod stiffness; PWS, partition wall stiffness.

The optimal values of LCRS and PWS depend on the maximum pressure, denoted by ${p_{max}}$, as well as the outer wall characteristics. Increasing the LCRS improves ${S_1} / {S_2}$, and thus, the design principles indicate that it increases performance, as qualitatively shown in Figure 10 (left). However, it requires a high PWS to prevent buckling, and therefore, the cross-sectional deformation can be compromised, which can reduce performance. Conversely, lower PWS facilitates the cross-sectional deformation, which according to the design principles is desirable, leading to higher initial deflections and higher performance at lower pressures, as qualitatively shown in Figure 10 (left). However, the maximum LCRS is then limited, which can reduce performance at higher pressures. A compromise is therefore necessary, which depends on ${p_{max}}$. The performance of designs optimized for different ${p_{max}}$ is qualitatively illustrated in Figure 10 (right), elucidating the fact that the optimal values of the parameters must be selected for the operating pressure in each scenario.

Since the cross-sectional deformation is limited by the outer wall, all designs become equivalent in terms of the cross section once the full cross-sectional deformation is reached. However, maximal PWS enables higher LCRS and therefore higher performance. In addition, high PWS also contributes to the longitudinal stiffness of wall 1, increasing ${S_1} / {S_2}$ and thereby leading to better performance. Thus, the optimization of the design involves selecting the maximum PWS that enables reaching the full cross-sectional deformation at ${p_{max}}$ and then the maximum LCRS to minimize tension at the outer wall during operation of the device while avoiding buckling. These parameters need to be optimized for each specific scenario. FE simulations were developed in this work for the optimization in the prototypical scenario. The specific simulations, the optimization process, and results obtained are reported in the section FE Simulations.

It should be noted that, in the design selected, the PWS serves to prevent buckling before the full cross-sectional deformation. After reaching the full cross-sectional deformation, this cross section remains practically constant despite further increases in pressure, and buckling does not occur since the contribution of the contracting effect is practically completed. Thus, designs with different PWS become equivalent once they reach the full cross-sectional deformation, provided that the rest of the design is equal and that the contribution of the partition walls to the longitudinal stiffness is relatively low.

## FE Simulations

FE simulations were developed to optimize the design parameters for the device in the prototypical scenario and verify the design principles extracted in the previous sections. The criteria to evaluate the performance are introduced in the Evaluation Criteria section, the discussion of the optimization process is presented in the Parameter Optimization section, the implementation of the FE simulations is detailed in the Simulation Implementation section, and the results are reported in the Simulation Results section.

### Evaluation criteria

**FIG. 11.**
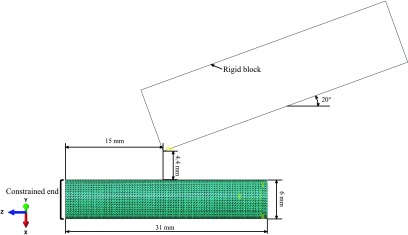
Configuration of the simulations with soft robotic manipulator and rigid block. The distances and angles are specified in the diagram.

The criteria to evaluate the performance of the soft robotic manipulator deserve consideration. The design objective in this prototypical scenario is to maximize the lateral force at a deflection near 20° for a given ${p_{max}}$. Thus, the performance is evaluated by measuring the normal force applied onto a prismatic block positioned, as shown in Figure 11, with frictionless contact. This corresponds to an approximate deflection near 20° of the manipulator at initial contact and an interaction that is normal to the rigid block and approximately lateral on the soft robotic manipulator. It should be noted that the deflection at initial contact is somewhat lower than 20°. This is intentional since the relative rotation between the ends of the manipulator varies with pressure even after contact, which implies that the distance between the center of the distal end of the device and the block changes even after contact. Since deflection is measured based on the position of the centers of the manipulator's ends, this varies at different pressures during contact. Thus, the rigid block is specifically positioned so that deflection is near 20° for the range of pressures of interest.

This configuration selected for the simulations is also a representative of the typical operation of soft robotic manipulators. The design principles were shown to be independent of maximum pressure and deflection. Thus, the FE simulations conducted in this configuration also serve to verify some of the design principles derived in the previous sections (Design of Extending Devices; Design of Contracting Devices; and summarized in section Summary).

### Parameter optimization

The objective of the optimization of the LCRS and PWS is to obtain both maximum PWS while reaching the full cross-sectional deformation at ${p_{max}}$ and minimal outer wall tension in the operation range of the device, while preventing buckling due to compression of the outer wall. This maximizes the forces that can be supported at ${p_{max}}$ and enables operation at low deflection.

The procedure to determine the optimal values of LCRS and PWS is as follows. First, PWS is selected to obtain the full cross-sectional deformation at ${p_{max}}$ for a generic LCRS. This is achieved by conducting quasistatic simulations for a set of values of PWS with regular stiffness increments while maintaining constant material properties elsewhere. The simulations are executed using a gradual increase in pressure until a practically full cross-sectional deformation, which here is specified by the central rod reaching 70% of the radius, and the corresponding pressure is recorded. The PWS of the design that achieves a practically full cross-sectional deformation at a pressure closest to ${p_{max}}$ is selected. Then, for the optimal PWS, the LCRS to achieve minimal outer wall tension is determined. This is done by conducting simulations with the optimal PWS and gradually increasing value for LCRS, starting with a stiffness corresponding to that of Dragon Skin 10, until the outer wall stiffness is minimal. The LCRS that reaches minimal outer wall stiffness without buckling, together with the PWS to provide a practically full cross-sectional deformation at ${p_{max}}$, constitute the optimal design. It should be noted that this optimization process of determining the PWS first independent of the LCRS is possible since the cross-sectional deformation is relatively independent of the value of LCRS.

It should also be noted that rupture of the partition walls due to excessive strain is not considered since the maximum cross-sectional deformation is limited by the outer wall. The maximum possible extension of the partition walls is approximately double their initial length, which is significantly below the failure limit of typical rubbers. Similarly, the extension of the central rod is typically lower than double the initial length, which is also below the failure limit. Thus, the PWS and LCRS varied freely.

### Simulation implementation

The simulations were implemented using Abaqus/Standard (Simulia™; Dassault Systemes, Velizy-Villacoublay, France). The simulation set up involves the soft robotic manipulator and a rigid block situated, as shown in Figure 11. The geometry of the soft robotic manipulator is a 6-mm-diameter cylinder, with a constant cross section as shown in Figure 13 (left), a solid end cap of 1 mm thickness, and a total length of 31 mm.

The material of the outer wall was modeled as an incompressible hyperelastic material with a Neo-Hookean constitutive law with *c*_10_ = 42,500 Pa and $D = 0$, following Ref.^33^ The constitutive behavior of the material of the partition walls was also approximated with an incompressible Neo-Hookean law, and the different values of the PWS were selected by varying the parameter ${c_{10}}$, with values ranging between *c*_10_ = 42,500 Pa and *c*_10_ = 425,500 Pa at regular increments of 42,500 Pa. Similarly, the material of the central rod was also approximated with an incompressible Neo-Hookean law, with a ${c_{10}}$ that was modified to vary LCRS. The bending stiffness of the central rod was not relevant at the stiffness values of interest since this was sufficiently thin. Finally, the fibers were modeled as circular beams of 10 μm diameter made of a material with a Young's modulus of 51 GPa, and a Poisson ratio of 0.36, which is representative of Kevlar.

An encastre boundary condition was imposed at one end of the manipulator, and another encastre was defined at one point of the rigid block. The contact between the manipulator and the rigid block was modeled as frictionless. The contact force was measured as the force applied by the manipulator on the rigid block.

**FIG. 12.**
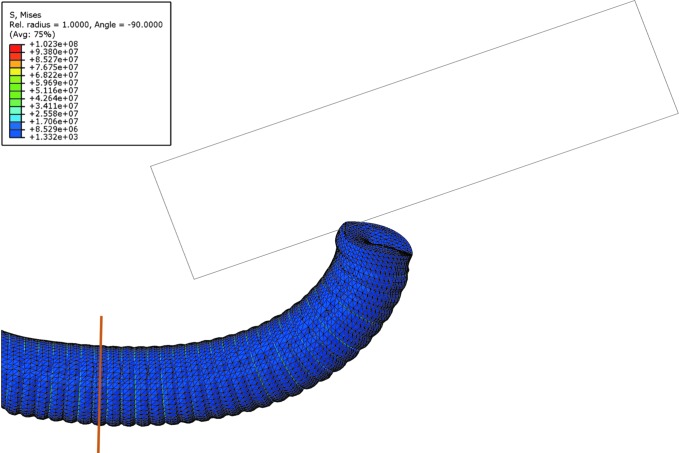
Simulation of the deformed geometry in design with partition walls made of a material with *c*_10_ = 127,500 Pa and central rod made of a material with *c*_10_ = 425 MPa, pressurized at 6 psi in two chambers. The bulging at the outer wall between the fibers is shown, as well as the deformation of the cap caused by the tension of the central rod.

The force corresponding to wall 2, *T*_2_, was measured as the aggregated tension force over the outer wall of the device in a free body cut corresponding to the cross section indicated in Figure 12. This is due to the fact that the outer wall in this 3D design provides the equivalent function as wall 2 in the analytical derivation. The mesh was maintained constant when varying material properties in the different simulations, and mesh convergence testing was conducted to ensure that the analysis was not affected by the characteristics of the mesh.

### Simulation results

The results of the simulations provide the deformation of the device and the force it applies on the rigid block as a function of pressure, as illustrated in Figure 12 for a representative simulation. These results serve both to determine the optimal design parameters of the device in the prototypical scenario and to verify some of the design principles.

#### Design optimization results

**FIG. 13.**
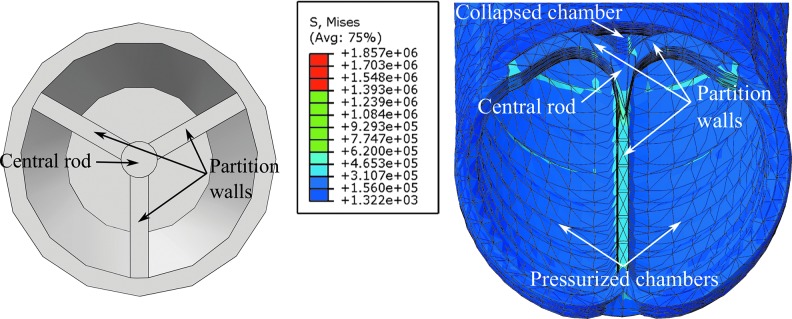
Undeformed cross-sectional geometry of the design selected viewed with a perspective projection (*left*), and deformed cross section of the same design with partition walls made of a material with *c*_10_ = 127,500 Pa when pressurized at 6 psi in two chambers (*right*).

In terms of optimal parameters, the results of varying PWS for constant LCRS show that partition walls made of a material with *c*_10_ = 127,500 Pa yield a practically full cross-sectional deformation at ${p_{max}}$, as shown in Figure 13 (right). This cross section corresponds to the section marked in orange in Figure 12, which is representative of the cross-sectional deformation along the device. Thus, the optimal PWS in this scenario corresponds to *c*_10_ = 127,500 Pa since it is the highest PWS that reaches a practically full cross section deformation at ${p_{max}}$.

**FIG. 14.**
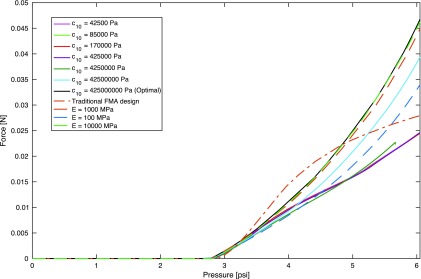
Plot of lateral force as a function of pressure for different designs. The performance of designs with a PWS of *c*_10_ = 127,500 Pa and a hyperelastic LCRS, the value of which is indicated in the legend in terms of the ${c_{10}}$ parameter, are shown in *continuous lines*. The performance of designs with a PWS of *c*_10_ = 127,500 Pa and an elastic LCRS, with the stiffness indicated in the legend in terms of Young's modulus, are shown in *dashed lines*. The performance of the traditional FMA is shown with an *orange line* combining *dots* and *dashes*. FMA, flexible microactuator.

**FIG. 15.**
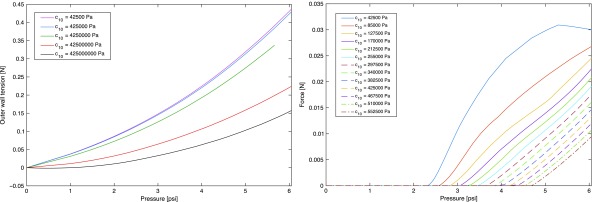
(*Left*) Plot of the outer wall tension as a function of pressure (*left*) for designs with a PWS of *c*_10_ = 127,500 Pa and various LCRS, indicated in the legend in terms of the ${c_{10}}$ coefficient. (*Right*) Plot of lateral force as a function of pressure for designs with varying PWS and equal material properties in the rest of design, including LCRS. The PWS are indicated in the legend in terms of the ${c_{10}}$ parameter of the Neo-Hookean constitutive law used to model these hyperelastic incompressible materials.

The results of increasing LCRS for the optimal PWS are shown in Figure 14. As can be seen, the performance improves with increasing values of LCRS. At an LCRS of *c*_10_ = 425 MPa, the tension at the outer wall becomes zero and even slightly negative during operation, as shown in Figure 15 (left), where the tension at the outer wall is plotted as a function of pressure for the different LCRS. Thus, the optimal LCRS corresponds to *c*_10_ = 425 MPa since higher values of LCRS would involve compression stress at the outer wall, which could lead to structural instabilities such as buckling. This was confirmed by executing simulations at higher LCRS, which presented converge issues due to structural instabilities.

A design with partition walls made of a material with *c*_10_ = 127,500 Pa and central rod with *c*_10_ = 425 MPa therefore represents optimal design in this scenario, together with the aforementioned geometry and the outer wall made of Dragon Skin 10 with *c*_10_ = 42,500 Pa. The higher performance of the optimal design is predominantly due to two factors. First, it presents a practically full cross section deformation at ${p_{max}}$, and therefore, it provides a high performance in terms of cross section as the area corresponding to the pressurized fluid is maximized and the majority of the stiffness is concentrated near the cross section contour corresponding to wall 1. Second, it has the highest LCRS, and therefore, the force spent stretching the structure is minimized, particularly at the central rod, which corresponds to wall 1, leading to a maximal contribution of pressure to support external forces. Interestingly, a relation can be observed between the reduction in tension at the outer wall, shown in Figure 15 (left), and the improvement in performance due to the increase in LCRS, shown in Figure 14, where the magnitude of the improvement in performance between two designs is directly related to the magnitude of the reduction in outer wall tension.

The results of the simulations show that buckling of the outer wall does not occur, as shown in Figure 12. The results also indicate that any bulging of the outer wall between the circumferential fibers is minor, as shown in Figure 12, which corresponds to a strain at the outer wall that is maintained significantly below the failure limit of the rubber. Thus, the wall performs as desired, withstanding the pressure applied without excessive wall thickness.

This outer wall behaves similarly to a pleated structure, presenting longitudinal extension with only minimal radial expansion between the fibers. Thus, this design with the soft outer wall and circumferential fibers is mostly equivalent to a previously mentioned design with a pleated structure and circumferential fibers, and the study developed here can be generally extrapolated due to the similar structural behavior.

The results of the simulations also confirm that the tension at the inner rod is relevant and significant. Observing the end cap, as shown in Figure 12, a depression can be noted, which is caused by the inner rod in tension.

#### Performance comparison

The performance of the design obtained in this work with optimal parameters was also compared with that of the FMA since it is a well-established design and is representative of some of the highest performing soft robotic manipulators that can meet the requirements of the scenario defined in this work. Dragon Skin 10 was selected as the material for the FMA to compare both designs in equivalent conditions. The results of lateral force as a function of pressure are shown in Figure 14. As can be seen, the design obtained here provides a higher force at ${p_{max}}$. The results also show that, at lower pressures, the FMA presents a somewhat higher performance, primarily due to the softer partition walls that enable larger cross-sectional deformation at lower pressure, confirming that a design must be optimized for a specific pressure.

#### Alternative materials

The design obtained in this work can be fabricated using readily available silicones for the outer wall, partition walls, and fibers. However, the hyperelastic material selected for the central rod can be difficult to obtain in practice as it presents stiffness significantly higher than that of standard rubbers. To consider more realistic materials, equivalent simulations were conducted using elastic material properties of the central rod, with Young's moduli between $E = {10^8}$ and $E = {10^{10}}$ Pa, which are representative of cotton or wool threads. The results are shown in Figure 14 together with the previous results for hyperelastic central rod. As can be seen, the performance of designs with central rods made of stiff elastic materials is equivalent to those with hyperelastic materials. Thus, the design can be fabricated by using readily available materials such as textile threads as the central rod.

#### Principles and operation verification results

The results of the simulations also serve to verify two of the most relevant design principles. In addition, they can be used to confirm that the operation of the device is as predicted.

The performance of different designs with varying PWS and constant LCRS and material properties elsewhere is plotted in Figure 15 (right) as a function of pressure. The plots indicate that lower PWS increases the lateral force that the device can apply and reduces the pressure required to attain an initial deflection. These results agree with the behavior predicted based on this analysis in this work, shown in Figure 10 (left). Thus, the results confirm that the cross-sectional deformation is desirable to improve the performance. Equivalently, the results verify that maximizing the area of the cross section corresponding to the pressurized fluid is desirable to maximize the force of soft robotic manipulators. This contrasts with some of the designs in the literature^19^ and shows that, unless additional constraints are present, such as those exposed in the Generalization to 3D section, the exploitation of the cross-sectional deformation can yield designs with improved performance.

The results of increasing values of LCRS with a constant design elsewhere, shown in Figure 14 for both hyperelastic and elastic central rods, confirm that increasing LCRS leads to higher force in general. These results also agree with the predicted trends, shown in Figure 10 (left). This verifies another of the design principles, namely that high LCRS is desirable to maximize the performance or, equivalently, that maximal ${S_1} / {S_2}$ is desirable to maximize the force that can be applied, provided that it does not lead to buckling of wall 2. It should be noted that the result of the simulation with a central rod stiffness of *c*_10_ = 4.25 MPa does not reach the full pressure. This is due to the fact that the simulation did not converge at pressures above 5.7 psi since some mesh elements presented excessive distortion. Nonetheless, the plot elucidates the trends of interest.

Finally, the results of tension at the outer wall for different LCRS, shown in Figure 15 (left), confirm that increasing LCRS leads to lower values of overall tension at the outer wall. Thus, these results confirm that the performance improves as less force is spent stretching the outer wall. In particular, for the optimal LCRS, the results in Figure 15 (left) show that the tension at the outer wall becomes zero and even to a slight extent negative, which indicate that the objective of the optimization in terms of minimizing outer wall tension is achieved. Moreover, the results on outer wall tension confirm that the contracting operation is effective, particularly at low deflections, where the tension at the equivalent of wall 2 becomes practically zero. At larger deflections, the contribution of the contracting operation is significantly reduced since the protrusion is limited by the outer wall and cannot increase further. Then, the extending operation becomes relevant, which involves some inevitable tension at the outer wall but provides a high overall performance.

## Conclusion

The design of soft robotic manipulators with fluidic actuation can be studied in general with the novel approach proposed in this work, which can serve as a common framework for the design of these devices. This approach can be first applied to justify the two main design layouts, which correspond to extending and contracting devices. Design principles for each of the two layouts can be subsequently extracted. In extending devices, the design should generally have minimal wall thickness to maximize the area of the cross section corresponding to the pressurized fluid; maximal cross section, using all available space; maximal longitudinal stiffness at the region of the cross section corresponding to one wall, with the stiffness concentrated near the contour in the direction of bending; and minimal longitudinal stiffness at the region of the cross section corresponding to the opposite wall. In contracting devices, the design should also generally have minimal wall thickness to maximize the area of the chambers in the cross section; a protruding wall with infinite longitudinal stiffness and a combination of minimal and infinite bending stiffness to maximize the increase in volume associated with an increment in contraction, using all available space; and another wall that can support compressive forces with minimum bending stiffness. In specific cases, contracting devices may include lateral walls, but these should only serve to contain the pressure, without affecting the structural behavior of the device.

The design principles for extending and contracting devices can be applied to determine the design of a soft robotic manipulator in a scenario of interest. To showcase this, a prototypical scenario was defined in this work. The application of the design principles led to the determination of the design of a soft robotic manipulator that combines the extending and contracting operation, which represents the most suitable design in the scenario defined. Optimal values for the stiffness of the partition walls and central rod in the design selected were found to require a numerical analysis of the deformation of the device. FE simulations were developed to determine these optimal stiffness values, yielding the optimized design. The FE simulations also served to confirm some of the main trends predicted by the design study, thereby verifying some of the main research results.
